# Untargeted metabolomic profiling of *Sphagnum fallax* reveals novel antimicrobial metabolites

**DOI:** 10.1002/pld3.179

**Published:** 2019-11-12

**Authors:** Jane D. Fudyma, Jamee Lyon, Roya AminiTabrizi, Hans Gieschen, Rosalie K. Chu, David W. Hoyt, Jennifer E. Kyle, Jason Toyoda, Nikola Tolic, Heino M. Heyman, Nancy J. Hess, Thomas O. Metz, Malak M. Tfaily

**Affiliations:** ^1^ Department of Environmental Science University of Arizona Tucson AZ USA; ^2^ Environmental Molecular Sciences Laboratory Pacific Northwest National Laboratory Richland WA USA; ^3^ Biological Sciences Division Pacific Northwest National Laboratory Richland WA USA; ^4^ Bruker Daltonics Inc Billercia MA USA

**Keywords:** antimicrobial metabolites, fungal metabolites, lipidomics, metabolomics, *Sphagnum fallax*

## Abstract

*Sphagnum* mosses dominate peatlands by employing harsh ecosystem tactics to prevent vascular plant growth and microbial degradation of these large carbon stores. Knowledge about *Sphagnum*‐produced metabolites, their structure and their function, is important to better understand the mechanisms, underlying this carbon sequestration phenomenon in the face of climate variability. It is currently unclear which compounds are responsible for inhibition of organic matter decomposition and the mechanisms by which this inhibition occurs. Metabolite profiling of *Sphagnum fallax* was performed using two types of mass spectrometry (MS) systems and ^1^H nuclear magnetic resonance spectroscopy (^1^H NMR). Lipidome profiling was performed using LC‐MS/MS. A total of 655 metabolites, including one hundred fifty‐two lipids, were detected by NMR and LC‐MS/MS—329 of which were novel metabolites (31 unknown lipids). *Sphagum fallax* metabolite profile was composed mainly of acid‐like and flavonoid glycoside compounds, that could be acting as potent antimicrobial compounds, allowing *Sphagnum* to control its environment. *Sphagnum fallax* metabolite composition comparison against previously known antimicrobial plant metabolites confirmed this trend, with seventeen antimicrobial compounds discovered to be present in *Sphagnum fallax*, the majority of which were acids and glycosides. Biological activity of these compounds needs to be further tested to confirm antimicrobial qualities. Three fungal metabolites were identified providing insights into fungal colonization that may benefit *Sphagnum*. Characterizing the metabolite profile of *Sphagnum fallax* provided a baseline to understand the mechanisms in which *Sphagnum fallax* acts on its environment, its relation to carbon sequestration in peatlands, and provide key biomarkers to predict peatland C store changes (sequestration, emissions) as climate shifts.

**Significance**

This work is significant because it illustrates using a comprehensive metabolomics approach, the potential mechanisms by which *Sphagnum fallax* inhibits microbial activity in peatlands and hence litter decomposition, leading to C sequestration. The identified metabolites provide a baseline to determine key biomarkers to predict peatland C store accumulation, sequestration, and emissions as climate shifts.

## 1 INTRODUCTION

Peatlands cover over 4 million km^2^ worldwide and are the most efficient terrestrial ecosystems in storing carbon (Gorham, 1991). The total amount of carbon stored in these systems is approximately 250–450 Pg C, equivalent to half of what is stored in the atmosphere as CO_2_ (750 Pg C; Frolking et al., 2011; Gorham, 1991). Northern peatlands are formed of bogs and fens, the former being a significant driver of organic matter accumulation, total amount of carbon stored, and total storage capacity (Armentano & Menges, 1986; Sjors, 1981). The bog arcotelm, defined as the peat thickness above the summer water table (Clymo, 1978), varies largely in the hummock/hollow microtopography, with a thicker layer of peat and correlative minimal C losses characteristic to hummocks (Wallén, 1986). This low organic carbon (OC) consumption and decomposition in peat bog hummocks is by and large due to the most important peat‐forming plants, the bryophytic moss *Sphagnum* (van Breemen, 1995; Verhoeven & Liefveld, 1997).

*Sphagnum* mosses, an ancient early‐branching lineage of land plants, are keystone species that shape their habitat through unique biochemical and morphological adaptations. Since these plants lack roots and sophisticated shoot systems for resource accumulation and distribution, *Sphagnum* mosses rely on employing hostile ecosystem tactics to ensure light, nutrient, and water availability (Kostka et al., 2016; Turetsky, 2018; van Breemen, 1995; Verhoeven & Liefveld, 1997). *Sphagnum* mosses have high capillarity and create permanently wet and water‐logged soil conditions, with resultant anoxia and relatively low temperatures. The characteristic cell‐wall structure, made of polysaccharides and highly reactive carbonyl groups, has been linked to a high‐cation exchange capacity which simultaneously traps nutrients and acidifies the environment (Ca concentration <2 mg/L and pH < 4.2; Clymo, 1978; van Breemen, 1995). As such, these adaptations wage environmental warfare on vascular plants and microbial degrading counterparts, which promotes *Sphagnum* growth, suppresses microbial and vascular plant growth, leads to subsequent slow litter (organic matter) decomposition, and ultimately results in the high carbon storage capacity observed in peat bogs.

Climate is one of the most important determinants of peatlands’ character, distribution, and biodiversity, yet peatland‐specific processes that control C decomposition, accumulation, and subsequent feedback to climate, despite their significance, are not included in current climate models (Limpens et al., 2008). Variability in primary productivity of peatlands is projected with increasing temperature, where some areas will benefit from lengthened growing seasons and concurrent warmer temperatures, and other areas will see a decrease in primary productivity due to evapotranspiration (Hufnagel & Garamvölgyi, 2014). A plausible shift from *Sphagnum* moss dominance to vascular plant species (i.e., sedges) is generally agreed upon in climate models, and because *Sphagnum* currently dictates and limits organic matter decomposition, in part by its restriction on microbial decomposer growth (and related organic matter degradation/greenhouse gas by‐products from microbial metabolism), these climates impacted shifts could affect the mobility of organic matter within peatland soils. While the understanding of individual biotic and abiotic drivers of C cycling in peatlands is developing (Chanton et al., 2008; Chasar, Chanton, Glaser, Siegel, & Rivers, 2000; Fenner & Freeman, 2011; Freeman, Evans, Monteith, Reynolds, & Fenner, 2001; Freeman, Ostle, & Kang, 2002), the plant–soil–microbe interactions in the context of rising temperatures remain poorly understood. This uncertainty in peatland response to climate change has therefore led to increase interest and urgency in better understanding the decomposition pathways of these large and important carbon stocks, and more specifically, the *Sphagnum* moss–microbial OM degradation interactions that underlay it.

While it is widely agreed upon that decomposition of peat carbon is partially inhibited by antimicrobial properties of *Sphagnum*, that is, by deterring the main soil decomposers, it is currently unclear which compounds are responsible for inhibition of organic matter decomposition and the mechanisms by which this inhibition occurs. Some researchers have suggested that the amorphous phenolic polymer that covers the surface of the *Sphagnum* cell wall, whose structure and function is similar to lignin, forms a physical barrier against decomposition (Freeman et al., 2001; Hájek, Ballance, Limpens, Zijlstra, & Verhoeven, 2011; Talbot & Treseder, 2012; Turetsky, Crow, Evans, Vitt, & Wieder, 2008; Verhoeven & Liefveld, 1997). However, Mellegård, Stalheim, Hormazabal, Granum, & Hardy, 2009 were unable to determine any antibacterial effects of phenolic compounds from moss extracts. Others have suggested that sphagnan, a pectin‐like compound within the cell‐wall holocellulose, is slowly released into the environment when the cell wall undergoes autogenic acid hydrolysis, liberating uronic acid for high‐cation exchange and acidification (Børsheim, Christensen, & Painter, 2001; Hájek et al., 2011; Painter, 1991; Stalheim, Ballance, Christensen, & Granum, 2009). The ambiguity in compounds and related function of various *Sphagnum* compounds on ecosystem variables elucidates an important research gap to gauge how these mosses control their ecosystems. Furthermore, this gap is widened by limitations in testing methodology, where previous studies have used singular analytical techniques which cannot identify a full suite of compounds (i.e., gas chromatography‐mass spectrometry, pyrolysis molecular beam mass spectrometry, ^13^C NMR; Black, Cornhill, & Woodward, 1955; Limpens, Bohlin, & Nilsson, 2017; Turetsky et al., 2008; Verhoeven & Liefveld, 1997), have focused only on specific compounds or classes of compounds (Ballance, Børsheim, Inngjerdingen, Paulsen, & Christensen, 2007; Klavina, Springe, Steinberga, Mezaka, & Ievinsh, 2018), or have concentrated on applied function rather than identifying structures (Bengtsson, Granath, & Rydin, 2016; Bengtsson, Rydin, & Hájek, 2018; Hájek et al., 2011). While all of these are crucial to understanding *Sphagnum* and its ecosystem roles, a full‐suite chemical characterization of *Sphagnum fallax* metabolites is currently missing from the literature.

Due to the importance of *Sphagnum* and the chemical structures underlying its dominance of peatland ecosystems, as well as the little available information of these structures and the general composition of *Sphagnum fallax*, this research aims to characterize *Sphagnum fallax* metabolite and lipid profile. We implemented a full‐analytical suite of mass spectrometry (MS) and ^1^H liquid state nuclear magnetic resonance spectroscopy (NMR) characterization techniques in order to identify known and unknown compounds of *Sphagnum fallax* to expand on and improve current databases. Our research further aims to understand the antimicrobial activity of *Sphagnum* and better understand its microbial inhibition mechanisms by using Fourier‐transform ion cyclotron resonance mass spectrometry (FTICR‐MS) to compare metabolomes and identify antimicrobial compounds in *Sphagnum* against the metabolomes of nine medicinal plants with previously known antimicrobial and medicinal properties (Ahmed et al., 2016; Cowan, 1999; Oliveira‐Alves et al., 2017; Ortega‐Ramirez et al., 2014; Riaz, Zia‐Ul‐Haq, & Jaafar, 2013; Singh, Khanam, Misra, & Srivastava, 2011; Surjushe, Vasani, & Saple, 2009; Yang et al., 2012). This research will provide fundamental chemical and structural data to further bridge the knowledge gap of how *Sphagnum fallax* acts on its environment, with the peatland microbiome, and the role it plays in the vulnerability or resilience of peatland carbon stores to changing environmental conditions. This information can be further extrapolated upon to improve climate models by identifying environmental biomarkers for understanding and predicting carbon changes in peatlands.

## 2 MATERIALS AND METHODS

### 2.1 Sample site—*Sphagnum fallax*

The experimental site was the S1 bog in the Marcell Experimental Forest (MEF; N 47°30.4760; W93°27.1620), approximately 40 km north of Grand Rapids, Minnesota, USA. The bog surface is characterized by hummock and hollow microtopography with a relief of 10–30 cm between the tops of the hollows and the hummocks. For more detailed information about this site refer to Kolka (2011) and Tfaily et al. (2014). S1 bog vegetation consists of two dominant tree species, black spruce (*Picea mariana*) and larch (*Larix laricina*), with both hollow and hummock features. The bryophytes in hollows are mainly colonized by *Sphagnum fallax*. Three biological replicates of individual plants of *Sphagnum fallax* were collected during August 2017. Only green living plants were sampled: samples focused on the capitulum plus about 2–3 cm of green living stem. *Sphagnum* stems (phyllosphere) were cleaned from unrelated plant debris and frozen immediately on dry ice and shipped overnight to the laboratory.

### 2.2 Sample collection and preparation

The 14 plant species analyzed in the antimicrobial portion of this study are included in Table 1. The *Sphagnum* collection site and collection procedure are described above. Medicinal plants were obtained as dry, loose leaf from the Tucson Herb Store; seed from Food Conspiracy Coop; dry, loose leaf from Hippie Gypsy; and live, dry, or powder from distributors on Amazon (JMBamboo, Bixa Botanical, Kart It). Creosote and controls (i.e., plants that were randomly collected and may not specifically have antimicrobial compounds) were collected from five locations on the University of Arizona campus in 15 ml sterile tubes. In preparation for organic matter metabolite extraction, triplicates of all plant samples were lyophilized using a Labconco FreeZone, Benchtop freeze dryer for 24 hr to remove moisture from samples and ensure moisture homogeneity between plant species for starting weight and extraction.

**Table 1:** Plant species tested in antimicrobial comparison study

Testing group	Plant	Plant material type
*Sphagnum* sp.	*Sphagnum fallax*	Stems, capitulum
Medicinal plants	Chamomile (*Matricaria recutita*)	Flower, stems
	Senna (*Cassia angustifolia*)	Leaves
	Mullein (*Verbascum thapsus*)	Leaves, stems
	Chia (*Salvia hispanica*)	Seeds
	Rosemary (*Rosemarinus officinacis*)	Leaves
	Cinnamon (*Cinnamomum cassia*)	Bark
	Aloe (*Aloe vera*)	Stems
	Creosote (*Larrea tridentate*)	Leaves, stems
	Cannabidiol (CBD – *Hemp cannabis*)	Flower, stems, seeds
Controls	Virgin Palm (*Dioon edule*)	Leaves, stems
	Arizona Cypress (*Cupressus arizonica*)	Leaves, stems
	Fountain Grass (*Cenchrus setaceus*)	Leaves
	Mexican Flame (*Anisacanthus quadrifidus*)	Leaves, stems

### 2.3 Organic matter metabolite extraction

Extracts were prepared using an adjusted Folch extraction (Folch, Lees, & Sloane Stanley, 1957). Folch extractions are used to extract labile (polar and semipolar compounds), and therefore, the detection of terpenes from these extracts can be limited. Lyophilized triplicate samples were ground using a standard electronic coffee grinder, weighed into three equal weights (about 0.1–0.05 g), and each triplicate per sample was wetted with 200 µl of milliQ water. Extraction was performed on each sample by sequentially adding 2 ml MeOH, followed by a 5 s vortex; 4 ml CHCl_3_, followed by a 5 s vortex; sonication at 25°C for 1 hr (CPX3800 Ultrasonic Bath, Fisherbrand); addition of 1.25 ml of H_2_O, followed by a slight mix to achieve bi‐layer separation; and incubated at 4°C overnight. The top, aqueous layer (metabolite—polar) was pipetted off into 1 ml glass vials and stored at −80°C until FTICR‐MS (all samples) and ^1^H NMR (*Sphagnum* only) analysis. The bottom, chloroform layer (*Sphagnum* only—nonpolar) was dried down and stored in 50:50 methanol:chloroform until lipidomics analysis.

### 2.4 Metabolomic analysis by FTICR‐MS

A Bruker 9.4‐Tesla FTICR spectrometer located at University of Arizona was used to collect high‐resolution mass spectra of the organic matter in the aqueous extract by direct injection. A standard Bruker electrospray ionization (ESI) source was used to generate negatively charged molecular ions. Samples were then introduced directly to the ESI source. The instrument settings were optimized by tuning on a Suwannee River fulvic acid (SRFA) standard, purchased from International Humic Substances Society (IHCC). Blanks (HPLC grade methanol) were analyzed at the beginning and end of the day to monitor potential carry over from one sample to another. The instrument was flushed between samples using a mixture of water and methanol. The ion accumulation time (IAT) was varied to account for differences in C concentration between samples and varied between 0.03 and 0.05 s. One hundred and forty‐four individual scans were averaged for each sample and internally calibrated using an organic matter homologous series separated by 14 Da (CH2 groups). The mass measurement accuracy was <1 ppm for singly charged ions across a broad *m/z* range (100–1,200 m*/z*). The mass resolution was ~240 K at 341 m*/z*. The transient was 0.8 s. Data Analysis software (BrukerDaltonik version 4.2) was used to convert raw spectra to a list of *m/z* values applying FTICR‐MS peak picker module with a signal‐to‐noise ratio (S/N) threshold set to 7 and absolute intensity threshold to the default value of 100. Putative chemical formulae were then assigned using Formularity (Tolić et al., 2017) software, as previously described in Tfaily, Hess, Koyama, and Evans (2018). Chemical formulae were assigned based on the following criteria: S/N > 7 and mass measurement error < 1 ppm, taking into consideration the presence of C, H, O, N, S, and P and excluding other elements. To ensure consistent formula assignment, we aligned all sample peak lists for the entire dataset to each other in order to facilitate consistent peak assignments and eliminate possible mass shifts that would impact formula assignment. We implemented the following rules to further ensure consistent formula assignment: (a) we consistently picked the formula with the lowest error between predicted and observed *m/*z and with the lowest number of heteroatoms, and (b) the assignment of one phosphorus atom requires the presence of at least four oxygen atoms. The chemical character of thousands of peaks in each sample's ESI FTICR‐MS spectrum was evaluated using van Krevelen diagrams. Compounds were plotted on the van Krevelen diagram based on their molar H:C ratios (*y*‐axis) and molar O:C ratios (*x*‐axis; Kim, Kramer, & Hatcher, 2003). Van Krevelen diagrams provide a means to visualize and compare the average properties of organic compounds and assign compounds to the major biochemical classes (e.g., lipid‐, protein‐, lignin‐, carbohydrate‐, and condensed aromatic‐like). In this study, biochemical compound classes are reported as relative abundance values based on counts of C, H, and O for the following H:C and O:C ranges: lipids (0 < O:C ≤ 0.3 and 1.5 ≤ H:C ≤ 2.5), unsaturated hydrocarbons (0 ≤ O:C ≤ 0.125 and 0.8 ≤ H:C < 2.5), proteins (0.3 < O:C ≤ 0.55 and 1.5 ≤ H:C ≤ 2.3), amino sugars (0.55 < O:C ≤ 0.7 and 1.5 ≤ H:C ≤ 2.2), lignin (0.125 < O:C ≤ 0.65 and 0.8 ≤ H:C < 1.5), tannins (0.65 < O:C ≤ 1.1 and 0.8 ≤ H:C < 1.5), and condensed hydrocarbons (aromatics; 0 ≤ 200 O:C ≤ 0.95 and 0.2 ≤ H:C < 0.8; Tfaily et al., 2015, 2017). It is important to note that FTICR‐MS by direct injection does not differentiate between isomers (i.e., compounds with same m/z, same molecular formula but different structure).

A 21 Tesla Agilent FTICR mass spectrometer (MS) equipped with a Waters ultra‐performance liquid chromatography (UP‐LC) system, was used to collect tandem mass spectrometry (LC‐MS/MS) spectra of triplicate *Sphagnum* organic matter extracts in negative and positive ion modes. *Sphagnum* extracts were separated using a Zorbax C18 column (0.5 mm × 150 mm × 5 µm particle size). Samples were injected in a 10 μL volume on column and eluted with solvent A (5 mM aqueous ammonium formate) and solvent B (5 mM ammonium formate in mass spectrometry grade methanol) with a 60 min linear gradient from 5% to 95% B, followed by isocratic elution at 95% B for 10 min at a flow rate of 0.2 ml/min. The flow from the LC was coupled to the 21 T FTICR‐MS using a heated ESI source set to a capillary voltage of 3,500 V; sheath, auxiliary, and sweep gas flow rates were 12, 6, and 2 (arbitrary units); and ion transfer tube and vaporizer temperatures were 300°C and 75°C, respectively. Mass spectra were collected with 1–2‐s transients. MS/MS fragmentation spectra were collected by collision‐induced dissociation (CID) of the major features using a collision energy of 40. Data were processed using Compound Discoverer 3.0 Software (Thermo Fisher Scientific—US) a qualitative data‐processing application that uses accurate mass data, isotope pattern matching, and mass spectral library searches for the structural identification of small molecules. The complete workflow is summarized in Figure S1. Briefly, we performed spectral library and similarity searches to identify compounds, searched chemical databases for putative candidates, and annotated spectra with predicted fragmentation. During the process, spectra were first aligned followed by peak picking. Predicted elemental composition using the TrueComposition^TM^ algorithm was generated for all unknowns using: exact mass, isotopic pattern, fine isotopic pattern, and MS/MS data using the built in HighChem Fragmentation Library of reference fragmentation mechanisms. Only compounds with MS/MS data were used for the analysis (i.e., compounds with relatively high intensity needed to generate MS/MS profiles). Metabolite identification was then performed using both spectral libraries and compound databases: mzCloud (ddMS2; online spectral library with >2 million spectra) that searches the online mzCloud database of fragmentation scans by using the MW or predicted formulas when available, and ChemSpider (exact mass or formula—chemical structure database with >480 data sources, 59 million structures) that searches selected databases of MS1 scans by using the MW or predicted formulas when available. For predicted composition, identification is dependent on mass error, number of matched isotopes, number of missed isotopes, number of matched fragments, spectral similarity score between theoretical and measured isotope pattern, matched intensity percentage of the theoretical pattern, matched intensity percentage of the relevant portion of the MS, and finally matched intensity percentage of the MS/MS scan. The mass tolerance was set to 5 ppm. After metabolite identification, data were grouped into two groups: group 1 included metabolites that were confirmed by the online database mzCloud and either predicted composition and ChemSpider or both, whereas group 2 included compounds that were validated by predicted composition and/or ChemSpider only. It is important to note that metabolites that were identified by Chemspider are only putative identifications since it is based on mass alone. Only metabolites that were identified in the three biological replicates were reported.

### 2.5 Metabolomic analysis by 1H NMR

One of the methanol extracted samples prepared as described above was used for metabolite profiling and quantitation by ^1^H NMR analysis to characterize common metabolites involved in primary metabolism and get an estimate of their abundance in living plants. The one‐dimensional (1D) ^1^H NMR spectrum of the *Sphagnum* sample was collected in accordance with standard Chenomx sample preparation and data collection guidelines (Weljie, Newton, Mercier, Carlson, & Slupsky, 2006). The sample was vacuum dried then reconstituted in deionized H_2_O. Even though vacuum drying would remove the majority of methanol in the sample, traces of methanol might remain in the sample after drying. Thus, our reported methanol concentration might be an overestimation of the true concentration of methanol in the plant extract. water extract was then diluted by 10% (v/v) with 5 mM 2,2‐dimethyl‐2‐silapentane‐5‐sulfonate‐d6 (DSS) as an internal standard, and the dilutions were accounted for in the normalized concentrations reported. NMR data were collected using a Varian Direct Drive 600 MHz NMR spectrometer equipped with a 5‐mm triple resonance salt‐tolerant cold probe and a cold‐carbon pre‐amplifier. The 1D ^1^H NMR spectra of all samples were processed, assigned, and analyzed using Chenomx NMR Suite 8.3 that combines chemistry and advanced algorithms to identify and quantify metabolites in NMR spectra with an extensive library of spectral models of metabolites (https://www.chenomx.com). In contrast to other analytical methods, only one internal standard is necessary for NMR analysis. Here, quantification was achieved through the use of an internal standard 4,4‐dimethyl‐4‐silapentane‐1‐sulfonic acid (DSS) that was used as chemical shift references for the calibration of the NMR data (at δ 0.0 ppm) as well as an internal standard for quantitation where spectral intensities of metabolites were quantified relative to the DSS internal standard. Candidate metabolites present in each of the complex mixtures were determined by matching the chemical shift, J‐coupling, and intensity information of experimental NMR signals screened against the NMR signals of standard metabolites in the Chenomx library (along with HMDB and some custom metabolite additions totaling ~1,000 metabolites). A list of the average chemical shifts per identified metabolite is included in Table S1. NMR spectra were collected as described previously (Dalcin Martins et al., 2017), and the detection limit of the method was ~1 µM. Only metabolites above 1 µM were included in this study. All concentrations were limited to three significant figures only.

### 2.6 Lipidomics analysis by LC‐MS/MS

Lipids were analyzed by LC‐MS/MS in both positive and negative ESI modes on the triplicate *Sphagnum* samples using a linear trap quadropole (LTQ) Orbitrap Velos mass spectrometer (Thermo Fisher Scientific), as described in detail previously (Kyle et al., 2017). Lipid species were identified using the LIQUID tool (Kyle et al., 2017) followed by manual data inspection. Confidently identified lipid species were quantified using MZmine 2 (Pluskal, Castillo, Villar‐Briones, & Orešič, 2010), and the peak intensities were normalized by linear regression and central tendency (i.e., identifying a central or typical value for a probability distribution) using InfernoRDN. Lipids that were identified in all three biological replicates were reported.

### 2.7 Network analysis

Network analysis was used to identify potential antimicrobial compounds in FTICR‐MS data based on specific chemical transformations between the pairs of *m/z* values, that is, *m/z* values of compounds of known antimicrobial effect and those *m/z* of unknown compounds. We used Cytoscape (Shannon et al., 2003) with the MetaNetter 2.0 plug‐in (Burgess, Borutzki, Rankin, Daly, & Jourdan, 2017) to conduct the network analysis. The list of *m/z* values from the *Sphagnum* sample was imported into Cytoscape, and each *m/z* value was defined as a node as described previously in Longnecker and Kujawinski (2016). In these networks, nodes are connected to each other by edges, where the edges are defined as the mass difference between two *m/z* values resulting from a chemical transformation. For example, the gain or loss of CO_2_ (Δ*m* = 43.9898) between two compounds would be represented as two “nodes,” or the corresponding smaller and larger *m/z* values, connected by an edge named CO_2_. For each chemical transformation, there may be a series of connected *m/z* values and one *m/z* value may be connected to other *m/z* values by more than one transformation. In this study, we used a pre‐defined list of *m/z* values for use as edges adopted from Breitling, Ritchie, Goodenowe, Stewart, and Barrett (2006).

## 3 RESULTS

### 3.1 LC‐FTICR‐MS/MS and ^1^H NMR analysis of the *Sphagnum* methanol layer (metabolomics)

Primary and secondary metabolites were analyzed by liquid state ^1^H NMR and LC‐FTICR‐MS/MS. A total of 29 metabolites were identified by ^1^H NMR (Table 2), representing metabolites commonly found in plants and soils. These metabolites were broadly categorized into eight groups: amino acids, acids, sugars, amines, polyols, choline, alcohols, and aromatic compounds. Sugars and acids were the most abundant metabolites and represented the highest concentration of total measured metabolites, with ~3,360 µM and ~1,010 µM in total per 50 mg of *Sphagnum* in 1 ml of DI H_2_O (extraction efficiency ~ 15%, Tfaily et al. 2015, 2017), respectively. Fructose was the most abundant measured metabolite present in *Sphagnum* (sugar; 2,490 µM), followed by glucose (sugar; 796 µM), malate (acid; 555 µM), and glycerol (polyol; 254 µM). Alcohols and aromatics represented the lowest overall concentration in *Sphagnum* (7.6 µM and 9.3 µM in total), with methylamine (amine; 3.3 µM), and propylene glycol (polyol; 2.2 µM) measured at the lowest concentrations of present metabolites. The majority of these compounds are of known biological activity, as inferred from the KEGG database (https://www.genome.jp/), signifying metabolites that could act as biomarkers of *Sphagnum* metabolic pathways (Table S2).

**Table 2:** Primary metabolites (with KEGG compound ID) found in *Sphagnum fallax* detected by ^1^H NMR

*Sphagnum*	KEGG compound ID	Formula	Compound concentration^a^	Compound class
Dimethylamine	C00543	C_2_H_7_N	10.2	Amines
4‐Aminobutyrate	C00334	C_4_H_9_NO_2_	142	
Methylamine	C00218	CH_5_N	3.3	
Propylene glycol	C00583	C_3_H_8_O_2_	2.2	Polyols
Glycerol	C00116	C_3_H_8_O_3_	254	
O‐Phosphocholine	C00588	C_5_H_15_NO_4_P	3.8	Cholines
Choline	C00114	C_5_H_14_NO	43.0	
Quinone	C00472	C6H_4_O_2_	9.3	Quinones
Methanol	C00132	CH_4_O	7.6	Alcohol
Pyruvate	C00022	C_3_H_4_O_3_	6.0	Acids
Lactate	C00186	C_3_H_6_O_3_	12.0	
3‐Hydroxybutyrate	C01089	C_4_H_8_O_3_	12.1	
Acetate	C00033	C_2_H_4_O_2_	236	
Fumarate	C00122	C_4_H_4_O_4_	13.2	
Malate	C00711	C_4_H_6_O_5_	555	
Formate	C00058	CH_2_O_2_	175	
Isoleucine	C00407	C_6_H_13_NO_2_	27.5	Amino Acids
Phenylalanine	C00079	C_9_H_11_NO_2_	30.7	
Uridine	C00299	C_9_H_12_N_2_O_6_	30.0	
Leucine	C00123	C_6_H_13_NO_2_	45.2	
Valine	C00183	C_5_H_11_NO_2_	58.4	
Alanine	C00041	C_3_H_7_NO_2_	160	
Threonine	C00188	C_4_H_9_NO_3_	41.6	
Asparagine	C00152	C_4_H_8_N_2_O_3_	188	
Aspartate	C00049	C_4_H_7_NO_4_	210	
Glucose	C00031	C_6_H_12_O_6_	796	Sugars
Fructose	C00095	C_6_H_12_O_6_	2,480	
Sucrose	C00089	C_12_H_22_O_11_	26.5	
myo‐Inositol	C00137	C_6_H_12_O_6_	48.9	

a µM per 50 mg of Sphagnum in 1 ml of DI H_2_O (extraction efficiency 15%).

LC‐FTICR‐MS/MS was performed to further identify and characterize secondary metabolites against TrueComposition ^TM^ algorithm (Predicted Composition—only compounds with MS2 data), mzCloud (online fragmentation spectral library with >2 million spectra), and ChemSpider (exact mass or formula—chemical structure database with >480 data sources, 59 million structures). Due to the high‐resolution of the instrument, this technique allows for high sensitivity detection and greater ability to detect previously uncharacterized compounds. Furthermore, all LC‐FTICR‐MS/MS and ^1^H NMR data were cross‐checked against the Plant Metabolic Pathway Network (PMN—https://www.plantcyc.org) database of documented *Sphagnum fallax* chemical compounds to determine which detected metabolites were novel or known metabolites in *Sphagnum fallax*. Positive mode LC‐FTICR‐MS/MS identified a total of 234 compounds of which 54 were validated by Predicted Composition and ChemSpider, and 20 were validated by Predicted Compositions, and mzCloud, and/or ChemSpider (Table 3; Table S3). Acids and sugars (in particular flavonoid glycosides) dominated the chemical classifications of the validated compounds, in addition to triols, amides, cholines, antioxidants, and flavones/oids. Riboflavin, luteolin, and adenosine (and their derivatives) were the only metabolites of the 54 named compounds that were recorded in the PMN database, signifying 51 identified compounds previously not known to be present in *Sphagnum*. Out of the 20 compounds, 10 were associated with a KEGG ID and involved in diverse pathways including flavone and flavonol biosynthesis, flavonoid biosynthesis, biosynthesis of secondary metabolites, purine metabolism, sphingolipid metabolism, alpha‐linolenic acid metabolism, biosynthesis of antibiotics, biosynthesis of unsaturated fatty acids, aflatoxin biosynthesis, and glutathione metabolism, all of which play important roles in plant metabolism and in its control and integration and defense against pathogens. The remaining 160 compounds were not found using mzCloud, Predicted Compositions, ChemSpider, or the PMN database, and signify novel metabolites detected in *Sphagnum* not identified or characterized in previous literature or databases.

**Table 3:** Secondary metabolites identified by LC‐FTICR‐MS/MS (positive ion mode) with KEGG ID and validated by mzCloud and predicted composition and/or ChemSpider (in triplicate samples)

Class	Name	Formula	mzCloud Search	mzCloud score	Predicted Compositions	Chemspider	Molecular Weight	KEGG ID
Triol	2‐Amino‐1,3,4‐octadecanetriol	C_18_H_39_NO_3_	Full match	99.7	Full match	Full match	317.2	C12144
Amide	α‐Linolenoyl ethanolamide	C_20_H_35_NO_2_	Full match	75.5	Full match	Not the top hit	321.2	
Acid	9S,13R‐12‐Oxophytodienoic acid	C_18_H_28_O_3_	Full match	98.3	Full match	Not the top hit	292.2	
Acid	3‐(4‐Hydroxy‐5‐oxo‐3‐phenyl‐2,5‐dihydro‐2‐furanyl)propanoic acid	C_13_H_12_O_5_	Full match	67.2	Full match	Partial match	248	
Vitamin	Riboflavin	C_17_H_20_N_4_O_6_	Full match	98.8		Full match	376.1	D00050
Sugar	2‐(3,4‐Dihydroxyphenyl)‐5,7‐dihydroxy‐4‐oxo‐4H‐chromen‐3‐yl 6‐O‐β‐D‐xylopyranosyl‐β‐D‐glucopyranoside	C_26_H_28_O_16_	Full match	69.6		Full match	596.1	
Antioxidants in plants	L‐Glutathione oxidized	C_20_H_32_N_6_O_12_S_2_	Full match	99.3		Full match	612.1	C00127
Sugar	2‐(3,4‐Dihydroxyphenyl)‐5,7‐dihydroxy‐4‐oxo‐4H‐chromen‐3‐yl 6‐O‐β‐D‐xylopyranosyl‐β‐D‐glucopyranoside	C_26_H_28_O_16_	Full match	69.6		Full match	596.1	
Uronic acid	6‐O‐Methylscutellarin	C_22_H_20_O_12_	Full match	99.3			476	
Sugar	(2S,2'S,3R,3'R)‐7'‐(β‐D‐Glucopyranosyloxy)‐5,5'‐dihydroxy‐2,2'‐bis(4‐hydroxyphenyl)‐4,4'‐dioxo‐3,3',4,4'‐tetrahydro‐2H,2'H‐3,3'‐bichromen‐7‐yl β‐D‐glucopyranoside	C_42_H_42_O_20_	Full match	64.7			866.2	
Sugar	(1ξ)‐1,5‐Anhydro‐1‐[2‐(3,4‐dihydroxyphenyl)‐5,7‐dihydroxy‐4‐oxo‐4H‐chromen‐8‐yl]‐D‐galactitol	C_21_H_20_O_11_	Full match	91.1		Not the top hit	448.1	
Mold metabolite	Aflatoxin B2	C_17_H_14_O_6_	Full match	61.3		Partial match	314	C16753
flavonoid, antioxidant	Luteolin	C_15_H_10_O_6_	Full match	66.1		Partial match	286	C01514
Sugar	(1S)‐1,5‐Anhydro‐2‐O‐(6‐deoxy‐α‐L‐mannopyranosyl)‐1‐[5,7‐dihydroxy‐2‐(4‐hydroxyphenyl)‐4‐oxo‐4H‐chromen‐6‐yl]‐D‐glucitol	C_27_H_30_O_14_	Full match	93.8		Partial match	578.1	
Sugar	4‐(3,4‐Dihydroxyphenyl)‐7‐methoxy‐2‐oxo‐2H‐chromen‐5‐yl 6‐O‐[3,4‐dihydroxy‐4‐(hydroxymethyl)tetrahydro‐2‐furanyl]hexopyranoside	C_27_H_30_O_15_	Full match	83.5		Partial match	594.1	
Pigenin flavone glucoside	Vitexin	C_21_H_20_O_10_	Full match	92.6		Partial match	432.1	C01460
Nucleoside	Adenosine	C_10_H_13_N_5_O_4_	Full match	99.7	Not the top hit	Full match	267	C00212
Flavone	Hispidulin	C_16_H_12_O_6_	Full match	99.1	Not the top hit	Full match	300	C10058
Acid	α‐Linolenic acid^a^	C_18_H_30_O_2_	Full match	88.9	Not the top hit	Full match	278.2	C06427
Acid	9S,13R‐12‐Oxophytodienoic acid	C_18_H_28_O_3_	Full match	99.5	Not the top hit	Not the top hit	292.2	C12144

a Possible lipid isomer (fatty acid).

Negative mode LC‐FTICR‐MS/MS identified a total of 240 compounds of which 71 compounds were validated by Predicted Composition and ChemSpider, and 31 were compounds validated by mzCloud, Predicted Composition, and/or ChemSpider (Table 4; Table S4). Acids dominated the compounds characterized by negative mode, followed by sugars (flavonoid glycosides), lipids, esters (one fatty acid ester), and an antioxidant. Prostaglandin E2 and pheophorbide b (and their derivatives) were the only metabolites of the 71 named compounds that were recorded in the PMN database, signifying 69 identified compounds previously not known to be present in *Sphagnum*. Eleven of the compounds (with 3 isomers) were identified in KEGG database involved in diverse pathways including as follows: Arachidonic acid metabolism (an elicitor of the plant defense response to phytopathogens), biosynthesis of unsaturated fatty acids, linoleic acid metabolism (common in plant leaves), biosynthesis of secondary metabolites, fatty acid biosynthesis, diterpenoid biosynthesis, cutin, suberine and wax biosynthesis, and glutathione metabolism (plays an important role in plant metabolism and cell function). The remaining 138 compounds with partial or no matches signify novel metabolites present in *Sphagnum* not identified or characterized in previous literature or databases.

**Table 4:** Secondary metabolites identified by LC‐FTICR‐MS/MS (negative ion mode) with KEGG ID and validated by mzCloud and predicted composition and/or ChemSpider (in triplicate samples)

Class	Name	Formula	mzCloud Search	mzCloud score	Predicted Compositions	ChemSpider	Molecular Weight	KEGG ID
Sugar	3‐Hydroxy‐3,5,5‐trimethyl‐4‐(3‐oxo‐1‐buten‐1‐ylidene)cyclohexyl β‐D‐glucopyranoside	C_19_H_30_O_8_	Full match	52.7	Full match		432.19971	
Acid	(2α,3β,19α)‐2,3,19‐Trihydroxyolean‐12‐en‐28‐oic acid	C_30_H_48_O_5_	Full match	54.5		Partial match	488.35047	
Acid	(2β,5ξ,8α,9β,10α,13α,15α)‐15‐Hydroxy‐2‐{[2‐O‐(3‐methylbutanoyl)‐β‐D‐glucopyranosyl]oxy}kaur‐16‐ene‐18,19‐dioic acid	C_31_H_46_O_12_	Full match	63	Full match		610.32098	
Lipid	Prostaglandin E2^a^	C_20_H_32_O_5_	Full match	75.8		Full match	352.2251	C00584
Acid	Neochlorogenic acid	C_16_H_18_O_9_	Full match	82.1		Full match	354.09513	
Sugar	(1S)‐1,5‐Anhydro‐2‐O‐(6‐deoxy‐α‐L‐mannopyranosyl)‐1‐[5,7‐dihydroxy‐2‐(4‐hydroxyphenyl)‐4‐oxo‐4H‐chromen‐6‐yl]‐D‐glucitol	C_27_H_30_O_14_	Full match	89.3		Partial match	578.16412	
Acid	8(S)‐Hydroxy‐(5Z,9E,11Z,14Z)‐eicosatetraenoic acid	C_20_H_32_O_3_	Full match	90.9		Partial match	320.2352	C14748
Acid	(15Z)‐9,12,13‐Trihydroxy‐15‐octadecenoic acid	C_18_H_34_O_5_	Full match	92.7	Not the top hit		330.24069	
Sugar	(1ξ)‐1,5‐Anhydro‐1‐[2‐(3,4‐dihydroxyphenyl)‐5,7‐dihydroxy‐4‐oxo‐4H‐chromen‐8‐yl]‐D‐galactitol	C_21_H_20_O_11_	Full match	97.6		Not the top hit	448.10082	
Acid	Pinolenic acid	C_18_H_30_O_2_	Full match	99.3		Not the top hit	278.2247	
Acid/fatty acid	Arachidonic acid^b^	C_20_H_32_O_2_	Full match	99.8		Full match	304.24041	C00219
Acid	(2α,3β,19α)‐2,3,19‐Trihydroxyolean‐12‐en‐28‐oic acid	C_30_H_48_O_5_	Full match	54.5	Not the top hit	Partial match	488.35047	
Lipid autacoids	6α‐Prostaglandin I1^a^	C_20_H_34_O_5_	Full match	70.8	Full match	Partial match	354.24071	
Acid	8(S)‐Hydroxy‐(5Z,9E,11Z,14Z)‐eicosatetraenoic acid^a^	C_20_H_32_O_3_	Full match	77.5	Full match	Partial match	320.2352	C14748
Acid	14(Z)‐Eicosenoic acid^b^	C_20_H_38_O_2_	Full match	89.7	Full match	Partial match	310.28723	
Acid	Corchorifatty acid *F* (plant metabolite, an antioxidant, and an antifungal agent.)	C_18_H_32_O_5_	Full match	93.5	Full match	Not the top hit	328.22505	
Acid	16‐Hydroxyhexadecanoic acid^b^	C_16_H_32_O_3_	Full match	94.5	Full match	Full match	272.23518	C18218
Acid	11,12‐Epoxy‐(5Z,8Z,11Z)‐icosatrienoic acid^a^	C_20_H_32_O_3_	Full match	94.9	Full match	Not the top hit	320.2352	C14748
Acid	Oleic acid^b^	C_18_H_34_O_2_	Full match	95.1	Full match	Full match	282.25602	C01712
Acid	8Z,11Z,14Z‐Eicosatrienoic acid^b^	C_20_H_34_O_2_	Full match	98.3	Full match	Partial match	306.25593	C03242
Acid	cis‐7‐Hexadecenoic acid^b^	C_16_H_30_O_2_	Full match	98.7	Full match	Partial match	254.22466	C08362
Fatty acid ethyl ester	Ethyl myristate	C_16_H_32_O_2_	Full match	98.7	Full match	Not the top hit	256.24036	
Antioxidant in plants	L‐Glutathione oxidized	C_20_H_32_N_6_ O_12_S_2_	Full match	99.1	Full match	Full match	612.15245	C00127
Acid	Eicosapentaenoic acid^b^	C_20_H_30_O_2_	Full match	99.5	Full match	Partial match	302.22472	C06087
Acid	9(Z),11(E)‐Conjugated linoleic acid^b^	C_18_H_32_O_2_	Full match	99.7	Full match	Not the top hit	280.24036	
Acid	5‐(3,5‐Di‐sec‐butyl‐1‐cyclopenten‐1‐yl)‐5‐hydroxy‐3‐oxopentanoic acid	C_18_H_30_O_4_	Partial match		Full match	Not the top hit	310.21445	
Ester	2‐methyl‐1,1'‐(1,10‐decanediyl) ester	C_18_H_30_O_4_	Not the top hit		Full match	Full match	310.21454	
Acid	(E,Z)‐9‐Hydroperoxy‐10,12‐octadecadienoic acid^a^	C_18_H_32_O_4_	Partial match		Full match	Full match	312.23018	
Acid	5‐(3,5‐Di‐sec‐butyl‐1‐cyclopenten‐1‐yl)‐5‐hydroxy‐3‐oxopentanoic acid	C_18_H_30_O_4_	Not the top hit		Full match	Not the top hit	310.21445	

a Possible oxidative product/derivative of lipid.

b Possible lipid isomer (fatty acid).

### 3.2 LC‐MS/MS analysis of the chloroform layer (lipidomics)

**Figure 1:**
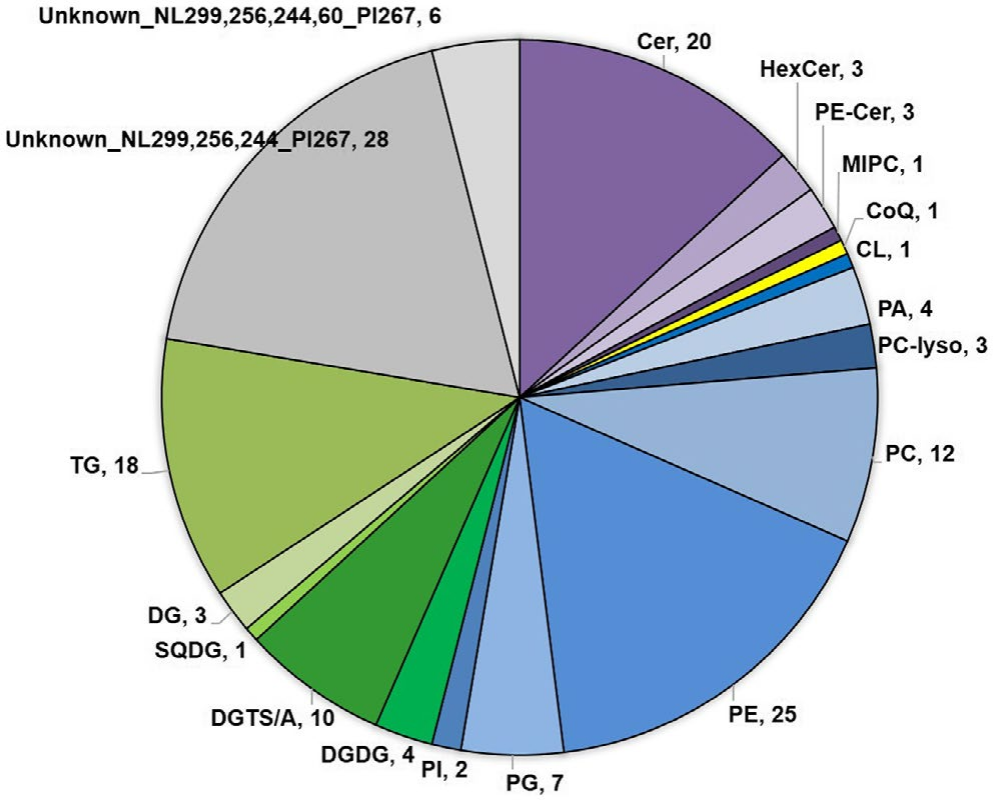
Distribution of identified lipids in *Sphagnum fallax* by subclass. Cer = ceramide, HexCer = galactose or glucose ceramide, PE‐Cer = ceramide phosphoethanolamine, MIPC = ceramide phosphoinositol, CoQ = coenzyme Q, CL = cardiolipin, PA = Diacylglycerophosphate, PC‐lyso = monoacylglcerophosphocholine, PC = diaoacylglcerophosphocholine, PE = diacylglcerophosphoethanolamine, PG = diacylglycerophosphoglycerol, PI = diacylglycerophosphoinositols, DGDG = digalactosyldiacylglycerol, DGTS/A = diacylglyceryl‐trimethyl‐homoserine or diacylglyceryl‐hydroxymethyl‐trimethyl‐alanine, SQDG = sulfoquinovosyldiacylglycerol, DG = diacylglycerol, TG = triacylglycerol. The number next to the lipid class abbreviation signifies the number of lipid species identified in that lipid class

One hundred and fifty‐two lipids were detected in *Sphagnum*, the majority of which were glycerophospholipid species, as well as 34 previously unidentified lipids (Figure 1, Table 5, Table S5). Polyunsaturated long‐chain fatty acids and glycerolipids have been broadly shown to be a naturally occurring group in bryophytic mosses (Mikami & Hartmann, 2004), and our data confirm their presence (Table 5). Previous studies on lipid composition of *Sphagnum* sp. have shown primarily alkyl compounds, sterols, triterpenoids, fatty acids, fatty alcohols, hydrocarbons, glycolipids, phospholipids, and some glycerophospholipids (Baas, Pancost, Geel, & Sinninghe Damsté, 2000; Klavina et al., 2018; Koskimies & Simola, 1980; Koskimies‐Soininen & Nyberg, 1987; Moore et al., 2015; Karunen & Ekman 1982). Our data confirmed the presence of sterols, fatty acids, prenyl derivative—ubiquinone, and glycerophospholipids, yet also demonstrated an abundance of sphingolipids and glycerolipids. Carotenoids and terpenes are also generally found in many plants (Tao, 2007) but were not detected in the lipidomics analysis of *Sphagnum fallax*.

**Table 5:** Lipid profile distribution of *Sphagnum fallax* by class and subclass as identified by LC‐MS/MS (positive and negative ion modes, in triplicate samples)

Lipid category	Subclass	# identifications
Sphingolipid	Cer	20
	HexCer	3
	PE‐Cer	3
	MIPC	1
Prenyl lipid	CoQ	1
Glycerophospholipid	CL	1
	PA	4
	PC‐lyso	3
	PC	12
	PE	25
	PG	7
	PI	2
Glycerolipid	DGDG	4
	DGTS/A	10
	SQDG	1
	DG	3
	TG	18
Unknown	Unknown_NL299,256,244_PI267	28
	Unknown_NL299,256,244,60_PI267	6
	Total identifications	152

### 3.3 Fungal metabolites and the presence of amine and nitrogen‐containing compounds

Three fungal metabolites were identified through positive mode LC‐FTICR‐MS/MS and confirmed using predicted compositions (matched by number and intensity of isotopes, and number and intensity of fragments) and ChemSpider (matched by mass): (8beta)‐6‐Allyl‐N‐[3‐(dimethylamino)propyl]ergoline‐8‐carboxamide, dehydroergosterol, and ergosterol (Table S6). Ergosterol is the predominant sterol of many fungi (ascomycetes and basidiomycetes) and is not naturally occurring in plants. Its concentration in plant samples is indicative of fungal growth and used to estimate fungal biomass associated with plant litter (Frainer & McKie, 2015; Gulis, Kuehn, & Suberkropp, 2009). Additionally, MIPC, a sphingolipid identified in the lipidomics data, is a fungal lipid commonly found in yeast (Kohlwein, 2017).

Thirty‐two nitrogen‐containing compounds were detected using LC‐FTICR‐MS/MS positive mode, which is greater than 60% of the identified compounds in this mode. Negative mode LC‐FTICR‐MS/MS detected 21 additional nitrogen‐containing compounds. ^1^H NMR analysis measured 4‐aminobutyrate (an amide) at a concentration of 142 µM, and while methylamine and dimethylamine were all also detected in 50 mg of *Sphagnum* per 1 ml of DI H_2_O by ^1^H NMR. Typically, boreal forests are nitrogen‐limited (Rousk, Jones, & DeLuca, 2013); however, this abundance of nitrogen could signify a nutrient pool available for nitrogen fixation in these ecosystems.

### 3.4 Acids and flavonoid sugars dominate the metabolome of *Sphagnum fallax*

LC‐FTICR‐MS/MS negative mode data show a surplus of acids present in *Sphagnum*, with 22 of the 31 validated compounds confirmed as acids, and seven more partial matches (Table 4; Table S4). Seven acid structures were confirmed via NMR, with acidic compounds present at the second highest total concentration in *Sphagnum* (1,010 µM/50 mg *Sphagnum*) following sugars (3,360 µM/50 mg *Sphagnum*; Table 2). Fittingly, *Sphagnum* moss is known to create an acidic environment in peatlands, which is suggested to be linked, in part, to *Sphagnum's* antimicrobial nature. Many sugar‐containing compounds (simple sugars and glucosides/glycosides), which can be “probiotic” and aid in microbial consumption and growth, were not only identified at highest concentrations in the NMR data but also confirmed in both LC‐FTICR‐MS/MS negative and positive mode. Fructose and glucose were measured at the highest concentrations of all identified compounds in the NMR characterization of *Sphagnum*, and 10 compounds were verified as sugar derivatives (flavonoid glycosides) via LC‐FTICR‐MS/MS.

### 3.5 Comparison of *Sphagnum* to medicinal plants using principal component analysis

**Figure 2:**
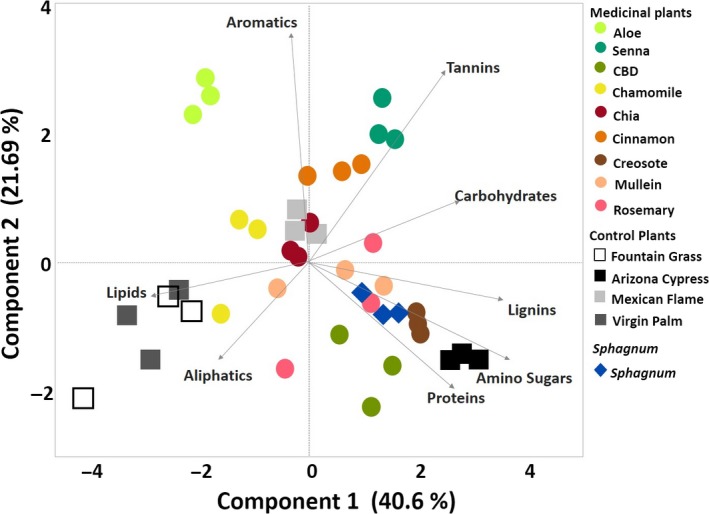
Principal component analysis plot derived from FTICR‐MS data (negative ion mode by direct injection), showing the distribution of the relative abundances of the different biogeochemical classes of compounds observed in each sample. For each plant, three biological replicates were analyzed

We used direct inject (DI) FTICR‐MS to compare *Sphagnum's* metabolite signature to the metabolite composition of different plant species with known antimicrobial effects (i.e., medicinal plants). Similarities and differences of chemical composition between medicinal, control, and *Sphagnum* were demonstrated using principal component analysis (Figure 2). *Sphagnum* showed greatest similarities across both components to mullein, creosote, CBD, and Arizona cypress. Lignin, amino sugars, and proteins were drivers of similarities between *Sphagnum* and like plants stated above. It should be noted that *Sphagnum fallax* is still driven by unique features, demonstrated by the separate clusters of each *Sphagnum* replicate plotted away from many of the other plant clusters, which signify there are likely distinctive metabolites that could serve as environmental biomarkers of *Sphagnum*.

### 3.6 Seventeen putative antimicrobial substances detected by FTICR‐MS in *Sphagnum*

To assess the antimicrobial similarities of chemical compounds between *Sphagnum* and medicinal plants, all *m/z* values from *Sphagnum fallax* were cross‐checked against a database of antimicrobial compounds confirmed in the FTICR‐MS analysis of the tested medicinal plants. The *m/z* of 17 known antimicrobial chemical compounds (Abedini et al., 2013; Akroum, Bendjeddou, Satta, & Lalaoui, 2009; Appendino et al., 2008; Bai et al., 2018; Chiruvella, Mohammed, Dampuri, Ghanta, & Raghavan, 2007; del Valle et al., 2016; Fiamegos et al., 2011; Huang, George, & Ebersole, 2010; Kandakumar & Manju, 2017; Lou et al., 2012; Mazimba, 2017; Nakatsuji et al., 2009; Narasimhan, Ohlan, Ohlan, Judge, & Narang, 2009; Sati, Dhyani, Bhatt, & Pandey, 2019; Shi et al., 2016; Singh, Sharma, Rani, Mishra, & Sharma, 2011) were confirmed to be present within *Sphagnum*. Two compounds had an associated isomer (Table 6); therefore, while 17 compounds were associated with antimicrobial traits medicinal, only 15 m*/z* values were detected. Of the 15 m*/z* values, five were detected in LC‐FTICR‐MS/MS positive and negative mode, including O‐glycoside‐luteolin and isomer kaempferol, O‐glycoside‐kaempferol and isomer luteolin glucoside, and caffeoylquinic acid. The absence of the 12 other compounds in the LC‐FTICR‐MS/MS data is likely that these compounds are present in low abundance, and hence, no MS/MS spectra were available during analysis of these ions in *Sphagnum*. *Sphagnum* showed the greatest similarities in antimicrobial qualities with chia, followed by chamomile and CBD. Of these antimicrobial compounds, nine of the seventeen are acids, which correlate with previous literature on *Sphagnum's* antimicrobial nature. Seven of these structures are sugar derivatives (e.g., flavonoid glycosides), which could be inhibiting microbial activity due to its antimicrobial properties. The presence of these putative antimicrobial compounds should be further tested via microbiological assays using *S. fallax* dominating strains to confirm the biological (antimicrobial) activity of these metabolites.

**Table 6:** Known antimicrobial compounds shared between *Sphagnum fallax* and the medicinal plants used in this study

Compound	Molecular formula	Plant associated with and confirmed
O‐glycoside‐aglycones apigenin	C_15_H_10_O_5_	CBD
O‐glycoside‐luteolin^a^, ^c^	C_15_H_10_O_6_	
Tetrahydrocannabinolic acid	C_22_H_30_O_4_	
O‐glycoside‐kaempferol^a^, ^d^	C_21_H_20_O_11_	
Umbelliferone	C_9_H_6_O_3_	Chamomile
Isorhamnetin	C_16_H_12_O_7_	
Luteolin glucoside^a^, ^d^	C_21_H_20_O_11_	
Quercetin glycoside	C_21_H_20_O_12_	
Coumaric acid	C_9_H_8_O_3_	Chia
Quinic acid	C_7_H_12_O_6_	
Syringic acid‐glucoside	C_15_H_18_O_9_	
Caffeoylquinic acid^a^	C_16_H_18_O_9_	
Methyl rosmarinic acid‐glucoside	C_18_H_16_O_8_	
Dodecanoic acid^b^	C_12_H_24_O_2_	Cinnamon
Palmitic acid ethyl ester	C_18_H_36_O_2_	Creosote
Veratric acid	C_9_H_10_O_4_	Mullein
Kaempferol^a^, ^c^	C_15_H_10_O_6_	Senna

a Compounds confirmed by LC‐FTICR‐MS/MS analysis.

b Possible lipid isomer—fatty acid 12:0.

c Isomers.

d Isomers.

### 3.7 Novel and known compounds identified by network analysis suggest potential pool of antimicrobial compounds present in *Sphagnum fallax*

**Figure 3:**
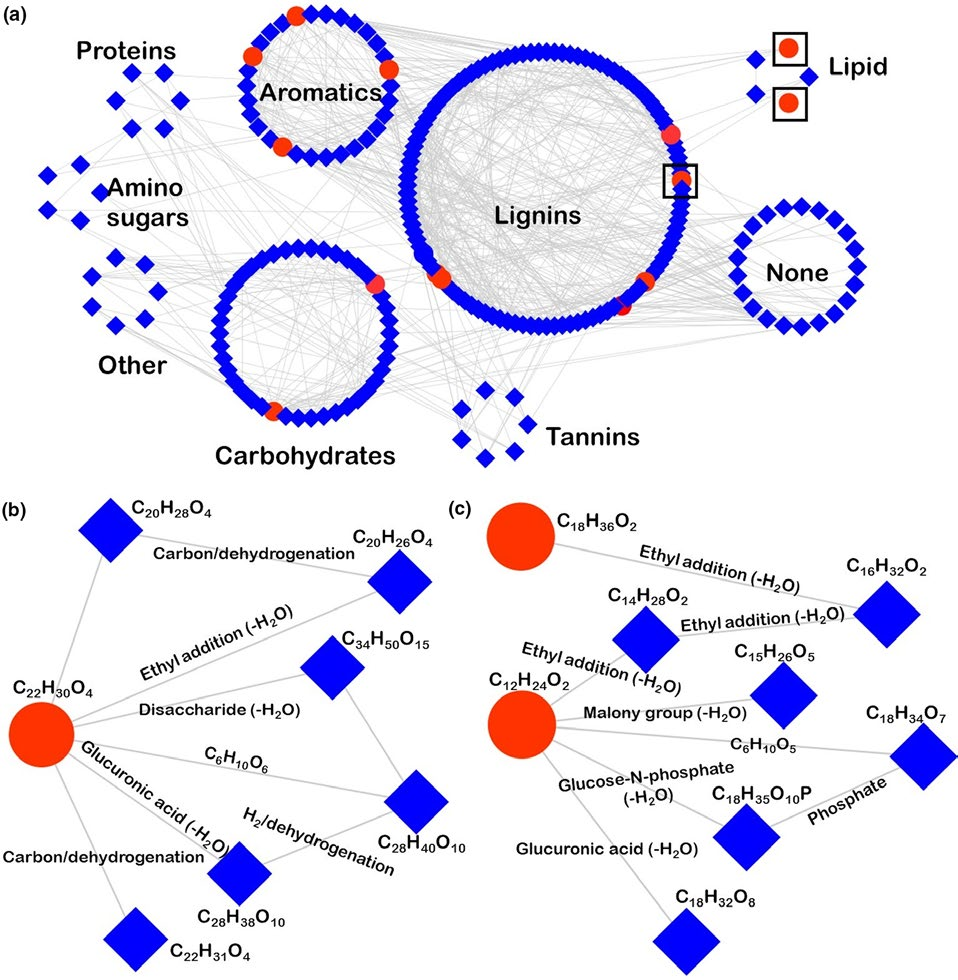
(a) Network analysis derived from FTICR‐MS (negative ion mode by direct injection) of the 17 antimicrobial compounds (dark purple circles) and 232 possible antimicrobial compounds (light purple squares) all detected in *Sphagnum fallax*. Possible antimicrobial compounds were determined by selecting first neighbors of the 17 identified antimicrobial compounds, that is, compounds that were one chemical reaction different between the pairs of *m/z* values (masses). Compounds are grouped by identified class, determined by oxygen:carbon and hydrogen:carbon ratios. Black squares represent the subnetworks have shown in b (polyphenols) and c (lipids) (b) Subnetwork of antimicrobial compound tetrahydrocannabinolic acid (circle) and all first neighbor compounds (squares) detected in *Sphagnum fallax*. (c) Subnetwork of antimicrobial compounds (circles) palmitic acid ethyl ester and dodecanoic acid, and all first neighbor compounds (squares) detected in *Sphagnum fallax*

Network analysis was performed on the potential antimicrobial substances present in *Sphagnum* to further identify known and unknown compounds that could be linked to *Sphagnum's* antimicrobial ability. In order to do so, all m/z values detected by DI‐FTICR‐MS in *Sphagnum* were analyzed and plotted by their differences in chemical transformations, and then, compounds were selected for that were first neighbors of (one transformation different from) the 17 putative antimicrobial metabolites. Compounds were submitted to Formularity software to generate formulas based on specific formula assignment rules, specifically assigning C, H, O, N, S, and P. Chemical classes were then determined by specific O:C and H:C ratios. Two hundred and thirty‐two masses were identified to be first neighbors of the 17 antimicrobial compounds, with 211 compounds that were assigned formulas and chemical classes (Table S7). For 21 of these compounds, no molecular formula was generated either because they contained elements outside of formula assignment rules or because they did not follow the rules used by Formularity. Therefore, these compounds are considered unknown. Polyphenol (lignin‐like) species dominate the antimicrobial and first neighbor compounds, followed closely by carbohydrate and aromatic compounds (Figure 3a). Two subnetworks are outlined in Figure 3b,c. Tetrahydrocannabinolic acid is one carbon/dehydrogenation from a previously unidentified species C_22_H_31_O_4_, which could suggest an undiscovered antimicrobial compound. Antimicrobial substances palmitic acid ethyl ester and dodecanoic acid are only three ethyl additions different, signifying the compounds C_16_H_32_O_2_ and C_14_H_28_O_2_ could also have similar antimicrobial effects within the environment. It should be noted that both C_16_H_32_O_2_ and C_14_H_28_O_2_ could be fatty acids 16:0 and 14:0, respectively, and each has 29 and 26 isomers. Further research should be done to structurally characterize these compounds and investigate their antimicrobial activity.

## 4 DISCUSSION

A comprehensive, state‐of‐the‐art approach to *Sphagnum fallax* metabolite characterization was used in order to further understand the *Sphagnum* metabolome and highlight the chemical compounds underlying *Sphagnum's* dominance of peatlands. By using a combination of ^1^H NMR, LC‐MS/MS, LC‐FTICR‐MS/MS, and DI‐FTICR‐MS to identify known and unknown compounds specific to *Sphagnum*, we were able to provide an extensive chemical characterization of *Sphagnum fallax* that is more in depth than previous literature (PMN Plant Cyc, *Sphagnum fallax*). A total of 655 metabolites, including 152 lipids, were detected by NMR and LC‐MS/MS—329 of which were novel metabolites including 31 unknown lipids. Acids, sugar, and their derivatives (flavonoid glycosides) dominated *Sphagnum* chemical composition. The lipid composition confirmed previous literature reports (Baas et al., 2000; Klavina et al., 2018; Koskimies & Simola, 1980; Koskimies‐Soininen & Nyberg, 1987; Moore et al., 2015) and also demonstrated a surplus of sphingolipids and glycerolipids. Given the high‐resolution data generated by these analytical techniques, this chemical profile of *Sphagnum fallax* has implications for identifying biomarkers that can dictate organic matter transformations in peatlands and help understand the mechanisms by which *Sphagnum* inhibits microbial activity.

The presence and importance of the fungal microbiome of *Sphagnum* sp. has been outlined in more recent years, with most of these fungal–moss relationships defined as parasitic and pathogenic toward *Sphagnum* (Kostka et al., 2016). Stenroos et al. (2010), however, showed a possible symbiotic relationship between *Sphagnum girgensohnii,* nitrogen‐fixing cyanobacteria *Nostoc*, and *Trizodia* fungi, where the fungus provided a less hostile environment for the cyanobacteria to reside and perform nitrogen fixation for the moss and fungi benefit. Our data identified three fungal metabolites, membrane lipid ergosterol (used to measure fungal growth), and its derivatives ((8beta)‐6‐Allyl‐N‐[3‐(dimethylamino)propyl]ergoline‐8‐carboxamide and dehydroergosterol), as well as one fungal sphingolipid, MIPC. We also identified 57 nitrogen‐containing compounds. Since nitrogen can be a limiting nutrient in peatlands (Rousk et al., 2013), our data suggest the possibility of *Sphagnum* metabolites as nitrogen sources and, furthermore, a possible moss–bacterial–fungal nitrogen fixation relationship due to the plethora of N‐containing compounds and ergosterol signifying fungal growth. More research should be conducted to characterize fungal species and the overall microbiome of *Sphagnum fallax* in this context.

One of the large factors in slow decay and carbon sequestration in peatlands is *Sphagnum's* ability to create a hostile environment and inhibit decomposer growth/decomposition. It has been suggested that phenolic compounds readily react with nutrients in the environment making them unavailable for decomposers, and the amorphous phenolic polymer that covers the surface of *Sphagnum* cell wall forms a physical barrier against decomposition (Freeman et al., 2001; Hájek et al., 2011; Talbot & Treseder, 2012; Turetsky et al., 2008; Verhoeven & Liefveld, 1997). Yet of all the compounds detected, our data only classified two compounds as phenols, 2‐{Bis[(4‐phenyl‐1,3‐thiazol‐2‐yl)amino]methyl}phenol and 4‐tert‐Octylphenol monoethoxylate, detected in LC‐MS negative mode (Table S3), and one quinone detected via ^1^H NMR at a concentration of 8.4 µM (Table 2). While these three could be the players in antimicrobial properties, it is likely these are not fully responsible for decomposer suppression, which corresponds with previous research (Bengtsson et al., 2018; Mellegård et al., 2009).

Acidification and nutrient sequestration is likewise suggested to be a player in the antimicrobial nature of *Sphagnum*, where highly reactive cation exchange (carbonyl/carboxyl) groups react with nutrient sources in the environment making them unavailable to decomposers and subsequently releasing protons into the environment lowering the pH to <4.2 (Børsheim et al., 2001; Hájek et al., 2011; Painter, 1991; Stalheim et al., 2009). This has mostly been attributed to Sphagnan, *Sphagnum* acid, and uronic acids in previous literature (Stalheim et al., 2009; Verhoeven & Liefveld, 1997). Our data support this trend, with more than 60% of structures identified by ^1^H NMR containing carbonyl/carboxyl functional groups, 1,010 µM of *Sphagnum* contributed to acids, and the discovery of thirty‐six acids via LC‐MS not previously known to be present in *Sphagnum*. Furthermore, in the comparison of *Sphagnum* to medicinal plants, greater than half (9 of 17) of the identified potential antimicrobial compounds were acids. Further research is needed to fully understand whether these acids are acting to control pH of peatlands, but it is likely these acidic, high‐cation exchange compounds are acting by suppression of microbial species.

Interestingly, *Sphagnum* makeup was also dominated by sugar‐like derivatives (glycosidases) and other complex sugars, which can be broken down by microbes into simple sugars (free glucose) and used for metabolism. However, 11 complex sugars were detected by LC‐FTICR‐MS/MS, and of the seventeen potential antimicrobial compounds present in *Sphagnum*, seven were also complex sugars derivatives. The detection of these complex sugars and their derivatives in *Sphagnum* are likely attributed to three phenomena. First, it is possible that the presence of acids and therefore low pH inhibits microbial growth and degradation of complex sugars for energy, which would account for both the detection of complex sugars and wealth of acids. Secondly, these complex sugars could be playing a role in inhibiting microbial growth, which has been shown in previous literature for these seven sugar derivatives in other plants (Akroum et al., 2009; Chiruvella et al., 2007; Sati et al., 2019; Shi et al., 2016; Singh, Sharma, et al., 2011; del Valle et al., 2016). Finally, network analysis identified 211 known compounds and 21 unknown compounds that were first neighbors of these 17 antimicrobial compounds, suggesting there are potentially other compounds in the *Sphagnum* metabolome that contribute to antimicrobial effects. Our data indicate that it is likely all three scenarios contributing to the antimicrobial nature of *Sphagnum*, which is crucial knowledge to understand biological factors underlying slow decay rates, peatland carbon sequestration, and how the shift from *Sphagnum* to vascular plants may act on or change this effect. With further research, the presence and intensity of these compounds could be used as biomarkers of antimicrobial activity in peatlands and indicate changes in carbon cycling and greenhouse gas fluxes as climate shifts.

Peatland terrestrial ecosystems are the largest carbon sinks worldwide, driven largely by Sphagnum's dominance over other plant species, control of slow decay rates and water table fluctuations, and subsequent changes in redox chemistry of peat bogs (Tfaily et al., 2014). The processes by which Sphagnum controls C decomposition, accumulation, and transformation are still poorly understood and, while relevant, are not included in current climate models. Once structural biomarkers are defined and linked to carbon specific processes, shifts in organic matter composition, and decomposition changes due to water table fluctuations, we can use these biomarkers to predict and anticipate peatland C transformations. With our high‐resolution mass spectrometry and liquid state NMR approach, we found 298 novel metabolites and few hundreds of compounds previously unknown to be present in Sphagnum fallax, which highlights the gap of knowledge on this species. Our research provides a much‐needed baseline characterization that can be extrapolated upon to understand the chemical structures (biomarkers) and their functions that underlay carbon sequestration and will aid in predicting what will happen in the face of climate shift, vascular plant dominance, and subsequent greenhouse gas fluxes.

## CONFLICT OF INTEREST

The authors declare no conflict of interest associated with the work described in this manuscript.

## AUTHOR CONTRIBUTIONS

MMT and JF wrote the manuscript with contributions from all coauthors. MMT, TOM, and HMH designed the experiment. RC, JT, DH, NT, HH, RA, JL, HG, and JEK collected and processed the data. MMT and JF conducted the analysis and interpretation. TM, NH, and MMT provided funding and guidance.
