# Supplementary material for: Untargeted metabolomic profiling of Sphagnum fallax reveals novel antimicrobial metabolites
Source: Plant Direct. 2019 Nov 12;3(11):e00179. doi: 10.1002/pld3.179 (PMC6848953; doi:10.1002/pld3.179)
Supplement: Supplementary file 1 [file PLD3-3-e00179-s001.pdf]

Supporting Information

Article title: Untargeted metabolic profiling of *Sphagnum fallax* from boreal peatlands identifies antimicrobial compounds and novel metabolites

Authors: Jane D. Fudyma[1], Jamee Lyon[1], Roya AminiTabrizi[1], Hans Gieschen[1], Rosalie Chu[2], David W. Hoyt[2], Jennifer E. Kyle[3], Jason Toyoda[2], Nikola Tolic[2], Heino M. Heyman[4], Nancy Hess[2], Thomas O. Metz[3], Malak M. Tfaily[1,2*]

Fig. S1 Complete workflow of LC/MS FTICR-MS data processing and analysis.

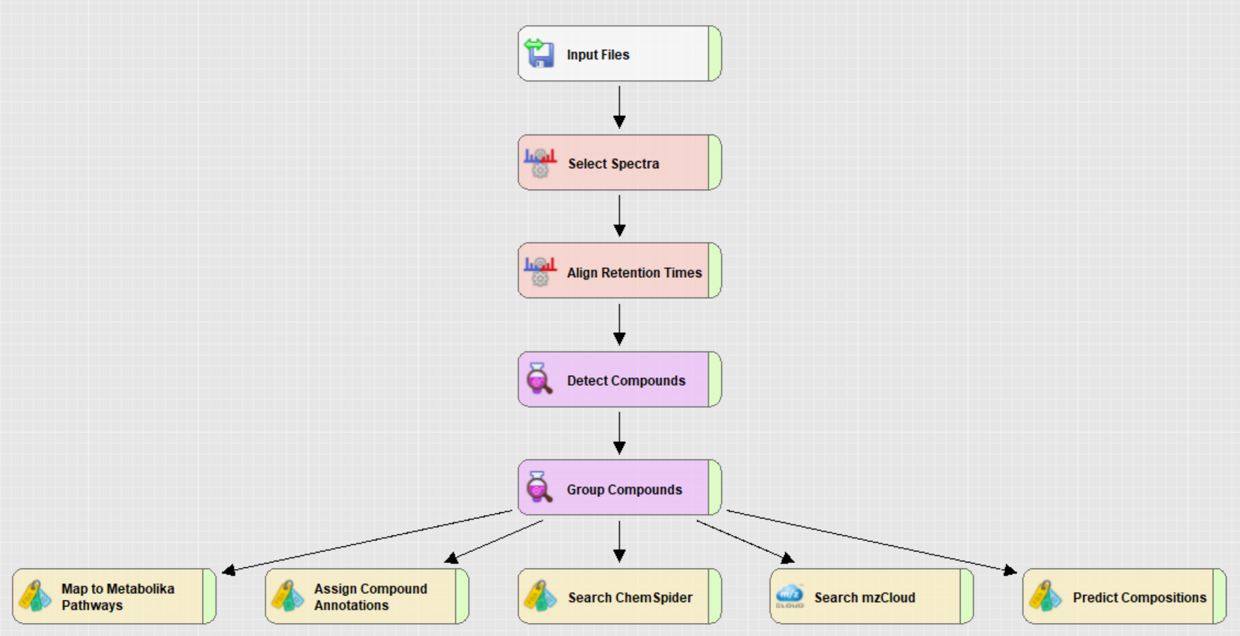

Table S1. A list of the average chemical shifts per identified metabolite is listed in Table is included in Table S1. m- multiplet, s- singlet, d, doublet, t-triplet, q- quartet, dd- doublet of doublets, dt-doublet of triplets

Metabolite	List of average chemical shifts (1H NMR)
3-Hydroxybutyrate	1.20(d)
4-Aminobutyrate	3.01(m), 2.30(t), 1.9(dt)
Acetate	1.95(s)
Alanine	3.78(q), 1.47(d)
Asparagine	4.00(dd), 2.95(dd), 2.85(dd)
Aspartate	3.89(dd), 2.82(dd), 2.67(dd)
Choline	4.06(m), 3.51(m), 3.19(s)
Dimethylamine	2.72(s)
Formate	8.44(s)
Fructose	4.11(dd), 4.10(dd), 4.01(dd), 3.99(m), 3.89(dd), 3.82(m), 3.79(dd), 3.78(d), 3.71(d), 3.70(dd), 3.67(dd), 3.59(d), 3.55(d), 3.54(d)
Fumarate	6.52(s)
Glucose	5.22(d), 4.64(d), 3.89(dd), 3.84 (m), 3.82(m), 3.76(dd), 3.72(dd), 3.71(m), 3.52(dd), 3.48(t), 3.45(m), 3.40(dd), 3.39(dd), 3.23(dd)
Glycerol	3.77(m), 3.64(dd), 3.55(dd)
Isoleucine	3.66(d), 1.97 (m), 1.45(m), 1.25(m), 1.00(d), 0.93(t)
Lactate	4.10(q), 1.32(d)
Leucine	3.73(dd), 1.74(m), 1.70(m), 1.67(m), 0.96(d), 0.95(d)
Malate	4.32(dd), 2.72(dd), 2.45(dd)
Methanol	3.35(s)
Methylamine	2.60(s)
O-Phosphocholine	4.16(m), 3.58(m), 3.21(s)
Phenylalanine	7.42(m), 7.37(ttt), 7.32(m), 3.99(dd), 3.28(dd), 3.11(dd)
Propylene glycol	1.13(d)
Pyruvate	2.36(s)
Quinone	6.80(s)
Sucrose	5.40(d), 4.21(d), 4.04((t), 3.88(m), 3.83(m), 3.82(dd), 3.82(d), 3.81(m), 3.80(m), 3.76(dd), 3.67(s), 3.66(s), 3.55(dd), 3.47(dd)
Threonine	4.25(dq), 3.58(d), 1.32(d)
Uridine	7.90(d), 5.90(d), 5.89(d), 4.34(dd), 4.20(t), 4.12(m), 3.90(dd), 3.80(dd)
Valine	3.60(d), 2.26(m), 1.03(d), 0.98(d)
myo-Inositol	4.05(t), 3.61(dd), 3.52(dd), 3.27(t)

Table S2. KEGG pathways associated with *Sphagnum* metabolism based on metabolites detected in *Sphagnum* via ^{1}H NMR. The number between parenthesis reflect the number of metabolites that mapped the pathway. Here we limited pathways that had at least 3 metabolites detected via ^{1}H NMR

KEGG Biological Pathway and ID	Metabolite Associated
ec01100 Metabolic pathways (27)	
ec01120 Microbial metabolism in diverse environments (15)	
ec01110 Biosynthesis of secondary metabolites (12)	
ec01130 Biosynthesis of antibiotics (11)	
ec00970 Aminoacyl-tRNA biosynthesis (8)	
ec00250 Alanine, aspartate and glutamate metabolism (6)	
ec00680 Methane metabolism (6)	
ec00052 Galactose metabolism (5)	
ec00290 Valine, leucine and isoleucine biosynthesis (5)	
ec00460 Cyanoamino acid metabolism (5)	
ec00620 Pyruvate metabolism (5)	
ec00260 Glycine, serine and threonine metabolism (4)	
ec00720 Carbon fixation pathways in prokaryotes (4)	
ec00760 Nicotinate and nicotinamide metabolism (4)	
ec00640 Propanoate metabolism (4)	
ec00966 Glucosinolate biosynthesis (4)	
ec00010 Glycolysis / Gluconeogenesis (4)	
ec00650 Butanoate metabolism (4)	
ec00261 Monobactam biosynthesis (3)	
ec00770 Pantothenate and CoA biosynthesis (3)	
ec00280 Valine, leucine and isoleucine degradation (3)	
ec00430 Taurine and hypotaurine metabolism (3)	
ec00630 Glyoxylate and dicarboxylate metabolism (3)	
ec00710 Carbon fixation in photosynthetic organisms (3)	
ec00270 Cysteine and methionine metabolism (3)	
ec00360 Phenylalanine metabolism (3)	
ec00500 Starch and sucrose metabolism (3)	

Table S3. Secondary metabolites identified by LC FTICR-MS/MS (positive ion mode) and validated by predicted composition and/or ChemSpider, where unknown compounds were generated a similarity compound based on mzCloud simulation. Compounds with a star are known fungal metabolites.

Class	Name	Formula	mzCloud Search	mzCloud Sim.	Predicted Compositions	ChemSpider	Molecular Weight	RT [min]
steroid alcohol*	Ergosterol	C28 H44 O			Full match	Full match	396.339	51.485
Dehydroergosterol*	Dehydroergosterol	C28 H42 O			Full match	Full match	394.324	50.675
ergoline alkaloids*	(8beta)-6-Allyl-N-[3-(dimethylamino)propyl]ergoline-8-Carbohydrateoxamide	C23 H32 N4 O		77.9	Full match	Not the top hit	380.256	41.587
Carbohydrateoxylate/Carbohydrateoxylic acid	2-Methyl-2-propanyl 4-fluoro-4-(hydroxymethyl)-1-piperidineCarbohydrateoxylate	C11 H20 F N O3		72.9	Full match	Not the top hit	233.142	30.951
Carbohydrateoxylate/Carbohydrateoxylic acid	2-[(Allyloxy)Carbohydrateonyl]-8-(tert-butoxyCarbohydrateonyl)-2,8-diazaspiro[4.5]decane-3-Carbohydrateoxylic acid	C18 H28 N2 O6		70.4	Full match	Not the top hit	368.197	37.068
Amine	3-(2-{2-[(2-Heptylundecyl)oxy]ethoxy}ethoxy)-1-propanamine	C25 H53 N O3			Full match	Full match	415.403	50.525
Acid	5-(Benzyloxy)-2-hexyl-3-hydroxyhexadecanoic acid	C29 H50 O4			Full match	Full match	462.371	55.441
Alcohol	1,1'-(Octadecylimino)dipropan-2-ol	C24 H51 N O2			Full match	Full match	385.392	49.481
stearate	253MC0P0YV	C24 H48 O6			Full match	Full match	432.345	45.707
Amino acid	Ethyl O-methyl-3-nitro-N-tetradecanoyl-L-tyrosinate	C26 H42 N2 O6			Full match	Full match	478.304	40.462
cholesterol ester	(-)-Cholesteryl acetate	C29 H48 O2			Full match	Full match	428.365	56.737
Carbohydrateamantes	Ethyl [5-amino-3-(4-aminophenyl)-1,2-dihydropyrido[3,4-b]pyrazin-7-yl]Carbohydrateamate	C16 H18 N6 O2			Full match	Full match	326.148	15.902
Amine	3-(2-{2-[(2-Heptylundecyl)oxy]ethoxy}ethoxy)-1-propanamine	C25 H53 N O3			Full match	Full match	415.403	49.512
Quinone	1,4-Bis{[2-(1-piperazinyl)ethyl]amino}-9,10-anthraquinone	C26 H34 N6 O2		89.4	Full match	Full match	462.275	49.866
cannabinoid	5-CHLORO-AB-PINACA	C18 H25 Cl N4 O2		62.2	Full match	Full match	364.165	34.33
nucleoside	Sdz-wag-994	C17 H25 N5 O4		89.6	Full match	Full match	363.189	12.592
Aromatic	3,4-Dihydro-2'H,11bH-spiro[phthalazino[2,1-a][3,1]benzoxazine-13,5'-pyrimidine]-1,2',4',6'(1'H,2H,3'H)-tetrone	C18 H14 N4 O5		77.3	Full match	Full match	366.095	25.586
Amine	5-Heptylbenzo[f]quinazoline-1,3-diamine	C19 H24 N4		80.3	Full match	Full match	308.199	38.037
Sugar	Methyl (5R)-5-[(1R,2R)-2-hydroxy-1-{[(4R)-1-methyl-4-propyl-L-prolyl]amino}propyl]-2,3-O-(oxomethylene)-1-thio-beta-L-arabinopyranoside	C19 H32 N2 O7 S		67.8	Full match	Full match	432.192	34.431
Aromatic	8-[4-(3,4-Difluorophenyl)-3-cyclohexen-1-yl]-1,4-dioxaspiro[4.5]decane	C20 H24 F2 O2		75.3	Full match	Full match	334.176	38.707
Amine	N-[1-(4-Benzyl-1-piperazinyl)-2-propanyl]-2-pyridinamine	C19 H26 N4		84	Full match	Full match	310.214	38.858
Other	Ethyl [(3-benzyl-4-oxo-4,6-dihydro-3H-spiro[benzo[h]quinazoline-5,1'-cyclohexan]-2-yl)sulfanyl]acetate	C28 H30 N2 O3 S		72.9	Full match	Full match	474.198	31.141
acetamide	Capsi-amide	C17 H35 N O		96.6	Full match	Full match	269.272	49.356
Other	Nipradilol	C15 H22 N2 O6		73	Full match	Full match	326.148	19.935
Amine	(E)-N-{2-[({5-[(Dimethylnitroryl)methyl]-2-furyl}methyl)sulfinyl]ethyl}-N'-methyl-2-nitro-1,1-ethenediamine	C13 H22 N4 O5 S		72.5	Full match	Full match	346.13	30.972
Benzene	2-(3,5-Dimethoxy-4-nitrophenoxy)-1,3,5-trimethoxy-4-nitrobenzene	C17 H18 N2 O10		61.8	Full match	Full match	410.098	26.199
Steroid	4,4-Difluoropregn-5-ene-3,20-dione	C21 H28 F2 O2		74.4	Full match	Full match	350.207	35.925
Quinone	5-O-methyl embelin	C18 H28 O4		87.6	Full match	Full match	308.199	37.327
	2210856	C18 H32 O2		82.6	Full match	Full match	280.24	50.663
Other	2,6-Diamino-5-[6-hydroxy-6-(1,3-oxazol-2-yl)hexyl]-4(1H)-pyrimidinone	C13 H19 N5 O3		95.4	Full match	Full match	293.147	2.814
Amine	4-[Bis(ethylsulfanyl)methyl]-N,N-dimethylaniline	C13 H21 N S2		87.1	Full match	Full match	255.111	4.963
	IN00216	C14 H13 N O4		78.7	Full match	Full match	259.084	18.496
	KJ9800000	C18 H39 O7 P		91.5	Full match	Full match	398.243	44.667
anthelmintic	1-BOC-4-(2-hydroxyethyl)piperazine	C11 H22 N2 O3		65.8	Full match	Full match	230.163	10.912
peptide	Leu-Leu	C12 H24 N2 O3		66.8	Full match	Full match	244.179	13.46
Other	3-(1-Phenyl-1H-pyrazol-5-yl)-1,2,3-benzotriazin-4(3H)-one	C16 H11 N5 O		84	Full match	Full match	289.095	10.256
Acid	tert-Butyl 2,7-diazaspiro[4.4]nonane-2-Carbohydrateoxylate	C12 H22 N2 O2		92.2	Full match	Full match	226.168	9.733
Unknown	[Similar to: (1S,2S,8ξ,9β,16α,17ξ)-2,16,20-Trihydroxy-9,10,14-trimethyl-11,22-dioxo-4,9-cyclo-9,10-secocholesta-5,25-dien-1-yl β-D-glucopyranoside; ΔMass: 198.2945 Da]			65	Full match		466.088	18.097
Unknown	[Similar to: Docosapentaenoic acid; ΔMass: -17.9144 Da]			72.5	Full match		348.17	38.445
Unknown	[Similar to: Chromomycin A3; ΔMass: 572.2663 Da]			75.5	Full match		610.243	48.711
Unknown	[Similar to: Eicosatetraynoic acid; ΔMass: -1.9501 Da]			63	Full match		298.128	2.426
Unknown	[Similar to: DAUDA; ΔMass: -174.0058 Da]			79.5	Full match		608.23	21.366
Unknown	[Similar to: (2S,3R,4R,5S,6E,14S)-4-Acetoxy-2-ammonio-3,5,14-trihydroxy-6-icosenoate; ΔMass: 54.0469 Da]			66	Full match		377.241	20.205
Unknown	[Similar to: MDMB-FUBINACA; ΔMass: -17.9863 Da]			69.3	Full match		415.167	16.557
Unknown	[Similar to: DAUDA; ΔMass: 22.0896 Da]			63	Full match		412.134	16.455
Unknown	[Similar to: Penta-N-acetylchitopentaose; ΔMass: 609.3279 Da]			67.4	Full match		424.08	20.524
Unknown	[Similar to: 6,18,19-Trihydroxytrachyloban-2-one; ΔMass: 0.0388 Da]			79.5	Full match		334.176	38.836
Unknown	[Similar to: 9-Nitrooleate; ΔMass: -23.9120 Da]			79.5	Full match		351.153	2.234
Unknown	[Similar to: Docosahexaenoic acid ethyl ester; ΔMass: -20.0415 Da]			68	Full match		376.313	49.537
Unknown	[Similar to: Nervonic acid methyl ester; ΔMass: 14.2209 Da]			76.9	Full match		366.145	34.348
Unknown	[Similar to: Perillartine; ΔMass: -99.0320 Da]			85.2	Full match		264.147	14.488
Unknown	[Similar to: 2,4-Bis(2,4-dimethylphenyl)-6-(2-hydroxy-4-n-octyloxyphenyl)-1,3,5-triazine; ΔMass: 244.1881 Da]			96.8	Full match		265.116	1.325
Unknown	[Similar to: Nervonic acid methyl ester; ΔMass: 14.1845 Da]			76.9	Full match		366.181	34.409
Unknown	[Similar to: Naringin dihydrochalcone; ΔMass: 123.0445 Da]			84.8	Full match		459.15	11.399
Unknown	[Similar to: N2-(2-Benzoylphenyl)-1-benzylpyrrolidine-2-Carbohydrateoxamide; ΔMass: -40.0008 Da]			76.6	Full match		424.185	18.107
Unknown	[Similar to: Dapsone; ΔMass: -171.8047 Da]			85.3	Full match		419.867	1.318
Unknown	[Similar to: 1-O-(1,3,23,24,25-Pentahydroxy-28-oxo-9,19-cyclolanostan-28-yl)hexopyranose; ΔMass: 270.2066 Da]			65.3	Full match		414.202	21.28
Unknown	[Similar to: AL 8810; ΔMass: 38.0344 Da]			67.7	Full match		364.186	32.418
Unknown	[Similar to: 15-Deoxy-Δ12,14-prostaglandin A1; ΔMass: 28.1040 Da]			70.9	Full match		290.115	25.55
Unknown	[Similar to: Linoleic acid-biotin; ΔMass: -27.8978 Da]			78.2	Full match		548.242	51.729
Unknown	[Similar to: Obacunone; ΔMass: 0.0902 Da]			62.4	Full match		454.109	1.343
Unknown	[Similar to: (2S,3R,4R,5S,6E,14S)-4-Acetoxy-2-ammonio-3,5,14-trihydroxy-6-icosenoate; ΔMass: 54.0469 Da]			64	Full match		377.241	18.843
Unknown	[Similar to: 4,4'-Methylenebis(3-chloro-2,6-diethylaniline); ΔMass: 137.0679 Da]			82.3	Full match		241.095	2.228
Unknown	[Similar to: Ergosterol peroxide; ΔMass: 196.3153 Da]			75	Full match		232.014	26.204
Unknown	[Similar to: (3β,5ξ,9ξ,16α,21β,22α)-28-Acetoxy-16,22,24-trihydroxy-21-{[(2E)-2-methyl-2-butenoyl]oxy}olean-12-en-3-yl β-D-glucopyranosyl-(1->2)-[β-D-glucopyranosyl-(1->4)]-β-D-glucopyranosiduronic acid; ΔMass: 522.2872 Da]			85	Full match		608.264	50.559
Unknown	[Similar to: Andrographiside; ΔMass: 144.0656 Da]			61.9	Full match		368.197	37.865
Unknown	[Similar to: Maslinic acid; ΔMass: -35.8581 Da]			79.7	Full match		508.213	21.615
Unknown	[Similar to: CAY10401; ΔMass: 0.1720 Da]			74.3	Full match		382.09	23.027
Unknown	[Similar to: (1R,2S,5aR,5bR,7aS,10R,12bR)-2-Hydroxy-10-isopropenyl-3,3,5a,5b,12b-			65.7	Full match		503.258	21.617

	pentamethyloctadecahydrodicyclopenta[a,i]phenanthrene-1,7a(1H)-diCarbohydrateoxylic acid; ΔMass: -16.9234 Da]				
Unknown	[Similar to: Linoleic acid-biotin; ΔMass: -27.8978 Da]	66	Full match	548.243	51.342
Unknown	[Similar to: AB-CHMINACA metabolite M3A; ΔMass: -18.0205 Da]	85.5	Full match	391.221	18.556
Unknown	[Similar to: 1-O-{3-[(6-Deoxyhexopyranosyl)oxy]-27-hydroxy-27,28-dioxoolean-12-en-28-yl}hexopyranose; ΔMass: 308.2535 Da]	78.3	Full match	486.192	34.259
Unknown	[Similar to: AB-CHMINACA metabolite M3A; ΔMass: -18.0205 Da]	89.5	Full match	391.221	18.44
Unknown	[Similar to: Haplamine; ΔMass: -96.0575 Da]	62.4	Full match	353.163	30.878
Unknown	[Similar to: 14(S)-HDHA; ΔMass: -83.9937 Da]	66.2	Full match	428.229	37.444
Unknown	[Similar to: Adenosine diphosphate (ADP); ΔMass: 97.9769 Da]	71.5	Full match	329.053	2.878
Unknown	[Similar to: 14(S)-HDHA; ΔMass: -120.0553 Da]	79.6	Full match	464.29	50.535
Unknown	[Similar to: Eicosapentaenoic acid methyl ester; ΔMass: 43.1553 Da]	95.8	Full match	273.085	1.806
Unknown	[Similar to: (2E,4E,9Z)-1-(piperidin-1-yl)hexadeca-2,4,9-trien-1-one; ΔMass: 21.1346 Da]	63.9	Full match	296.137	15.247
Unknown	[Similar to: Docosapentaenoic acid methyl ester; ΔMass: -17.8781 Da]	60.9	Full match	362.15	36.857
Unknown	[Similar to: AB-CHMINACA metabolite M3A; ΔMass: -17.9994 Da]	77.2	Full match	391.2	30.445
Unknown	[Similar to: 16,16-Dimethyl prostaglandin A1; ΔMass: -112.0291 Da]	75.6	Full match	476.29	50.781
Unknown	[Similar to: CAY10401; ΔMass: -328.1080 Da]	65.1	Full match	710.37	35.503
Unknown	[Similar to: Norsufentanil; ΔMass: -18.0357 Da]	82.3	Full match	294.22	37.432
Unknown	[Similar to: 1-O-[5-({5-[(3,5-Dihydroxydecanoyl)oxy]-3-hydroxydecanoyl}oxy)-3-hydroxydecanoyl]hexitol; ΔMass: 372.2593 Da]	63	Full match	368.197	37.462
Unknown	[Similar to: (4R,9β,23E)-2-(β-D-Glucopyranosyloxy)-16,20-dihydroxy-9,10,14-trimethyl-1,11,22-trioxo-4,9-cyclo-9,10-secocholesta-2,5,23-trien-25-yl acetate; ΔMass: 204.2087 Da]	63.5	Full match	514.148	29.326
Unknown	[Similar to: all-cis-4,7,10,13,16-Docosapentaenoic acid; ΔMass: 0.0751 Da]	66.3	Full match	330.181	42.119
Unknown	[Similar to: (6aR,9R,9aR)-9-Acetyl-3-[(1E,3E,5S)-3,5-dimethyl-1,3-heptadien-1-yl]-6a-methyl-9,9a-dihydro-6H-furo[2,3-H]isochromene-6,8(6aH)-dione; ΔMass: -328.1920 Da]	63.8	Full match	710.37	35.937
Unknown	[Similar to: Cortisol; ΔMass: -57.9515 Da]	80.4	Full match	420.161	18.829
Unknown	[Similar to: Morphine 6-β-D-glucuronide ; ΔMass: 158.0143 Da]	71.9	Full match	303.154	1.748
Unknown	[Similar to: 16,16-Dimethyl prostaglandin A1; ΔMass: -40.9750 Da]	91.1	Full match	405.236	23.724
Unknown	[Similar to: Enalapril; ΔMass: 50.9997 Da]	82.4	Full match	325.2	30.968
Unknown	[Similar to: Cortisol; ΔMass: -161.9051 Da]	60.2	Full match	524.114	1.85
Unknown	[Similar to: Sinapine; ΔMass: -12.9816 Da]	92.2	Full match	322.139	35.954
Unknown	[Similar to: Neosolaniol; ΔMass: -91.0032 Da]	88.6	Full match	473.166	12.219
Unknown	[Similar to: (4R,9β,23E)-2-(β-D-Glucopyranosyloxy)-16,20-dihydroxy-9,10,14-trimethyl-1,11,22-trioxo-4,9-cyclo-9,10-secocholesta-2,5,23-trien-25-yl acetate; ΔMass: 352.1755 Da]	80.7	Full match	366.181	35.504
Unknown	[Similar to: Maltopentaose; ΔMass: -184.0356 Da]	87.2	Full match	1012.31	2.55
Unknown	[Similar to: 2,4,6-Trideoxy-6-{[(4E)-3-hydroxy-2,4-dimethyl-4-heptenoyl]amino}-2,4-dimethylhex-5-ulosonic acid; ΔMass: 0.0728 Da]	86.4	Full match	343.127	2.505
Unknown	[Similar to: (2E)-2-({[(3aR,4R,6E,9S,10Z,11aS)-9-Acetoxy-6,10-dimethyl-3-methylene-2-oxo-2,3,3a,4,5,8,9,11a-octahydrocyclodeca[b]furan-4-yl]oxy}Carbohydrateonyl)-2-buten-1-yl (2E)-2-(hydroxymethyl)-2-butenoate; ΔMass: 145.0302 Da]	86.2	Full match	357.19	15.143
Unknown	[Similar to: Palmitoyl sphingomyelin; ΔMass: -17.9143 Da]	62	Full match	720.482	49.516
Unknown	[Similar to: Quinine; ΔMass: 0.0153 Da]	72.1	Full match	324.169	19.51
Unknown	[Similar to: 3-Methyl-5-(5,5,8a-trimethyl-2-methylene-7-oxodecahydro-1-naphthalenyl)pentyl acetate; ΔMass: -17.8763 Da]	86.4	Full match	366.143	10.205
Unknown	[Similar to: Methyl 5,6-diacetoxy-10-hydroxy-2,4b,7,7,10a,12a-hexamethyl-12-methylene-1,4,8-trioxohexadecahydro-2H-naphtho[1,2-H]isochromene-2-Carbohydrateoxylate; ΔMass: 80.9261 Da]	62.3	Full match	495.331	40.441
Unknown	[Similar to: 2-Hydroxy-22-(2-hydroxy-2-propanyl)-3,8,8,17,19-pentamethyl-23,24-dioxaheptacyclo[19.2.1.01,18 03,17 04,1407,12 012,14]tetracos-4-en-9-yl β-D-xylopyranoside; ΔMass: 150.0188 Da]	60.1	Full match	468.358	56.859
Unknown	[Similar to: Chromomycin A3; ΔMass: 587.3405 Da]	68.4	Full match	595.169	2.452
Unknown	[Similar to: Azithromycin; ΔMass: 140.2449 Da]	74.3	Full match	608.264	49.527
Unknown	[Similar to: 1,5-Anhydro-1-[5,7-dihydroxy-2-(4-hydroxyphenyl)-4-oxo-4H-chromen-6-yl]hexitol; ΔMass: -132.0424 Da]	85.3	Full match	564.148	23.839
Unknown	[Similar to: Methyl (2E)-3-[5-acetoxy-9-(3-furyl)-4-hydroxy-3,3,5a,9a-tetramethyl-7-oxododecahydro[2]benzofuro[5,4-f]oxireno[d]isochromen-11b(1H)-yl]acrylate; ΔMass: 164.1587 Da]	61.2	Full match	380.072	1.612
Unknown	[Similar to: Eicosapentaenoic acid methyl ester; ΔMass: 43.1424 Da]	95.8	Full match	273.098	1.656
Unknown	[Similar to: 9-Nitrooleate; ΔMass: 0.1091 Da]	95.7	Full match	327.132	5.424
Unknown	[Similar to: 1-[2-(Benzyloxy)phenyl]-2-(1H-imidazol-1-yl)-3-phenylprop-2-en-1-one; ΔMass: -161.9725 Da]	86.4	Full match	542.125	1.779
Unknown	[Similar to: 2-Hydroxy-3-(5-hydroxy-7,8-dimethoxy-4-oxo-4H-chromen-2-yl)phenyl β-D-glucopyranoside; ΔMass: -13.9793 Da]	95.2	Full match	506.106	26.47
Unknown	[Similar to: (S)-10-Hydroxycamptothecin; ΔMass: -17.9841 Da]	82.3	Full match	382.09	20.052
Unknown	[Similar to: 1,2-Dipalmitoylphosphatidylglycerol; ΔMass: 60.0632 Da]	60.9	Full match	662.447	56.587
Unknown	[Similar to: (3β,5ξ,9ξ,16α,21β,22α)-28-Acetoxy-16,22,24-trihydroxy-21-{[(2E)-2-methyl-2-butenoyl]oxy}olean-12-en-3-yl β-D-glucopyranosyl-(1->2)-[β-D-glucopyranosyl-(1->4)]-β-D-glucopyranosiduronic acid; ΔMass: 522.2872 Da]	89.2	Full match	608.264	51.129
Unknown	[Similar to: Linoleic acid-biotin; ΔMass: -71.9238 Da]	91	Full match	592.269	52.427
Unknown	[Similar to: Linoleic acid-biotin; ΔMass: -71.9238 Da]	89.5	Full match	592.269	52.15
Unknown	[Similar to: Philanthotoxin 74; ΔMass: -18.0106 Da]	90.5	Full match	452.336	22.341
Unknown	[Similar to: α-Linolenoyl ethanolamide; ΔMass: -17.9854 Da]	90.4	Full match	339.252	17.695
Unknown	[Similar to: 12-Benzyl-3-isobutyl-6,9-diisopropyl-5,8-dimethyldodecahydro-1H,3H-pyrrolo[1,2-q][1,4,7,10,13,17]oxapentaazacyclononadecine-1,4,7,10,13,17(14H)-hexone; ΔMass: -48.7835 Da]	78.8	Full match	704.178	1.957
Unknown	[Similar to: CAY10429; ΔMass: -15.8494 Da]	79.6	Full match	330.074	33.298
Unknown	[Similar to: Arachidonic acid ethyl ester; ΔMass: 28.0313 Da]	77.4	Full match	304.24	50.519
Unknown	[Similar to: Cortisol; ΔMass: -168.1914 Da]	78.4	Full match	530.401	58.656
Unknown	[Similar to: (4R,9β,23E)-2-(β-D-Glucopyranosyloxy)-16,20-dihydroxy-9,10,14-trimethyl-1,11,22-trioxo-4,9-cyclo-9,10-secocholesta-2,5,23-trien-25-yl acetate; ΔMass: 352.1755 Da]	80.7	Full match	366.181	35.937
Unknown	[Similar to: Cytosine; ΔMass: -114.0569 Da]	95.6	Full match	225.1	16.746
Unknown	[Similar to: Bergapten; ΔMass: -326.0615 Da]	91.8	Full match	542.104	20.024
Unknown	[Similar to: Hexadecanamide; ΔMass: -74.8993 Da]	73.3	Full match	330.156	30.966
Unknown	[Similar to: Hydrolyzed fumonisin B1; ΔMass: 0.1091 Da]	92.8	Full match	405.236	22.393
Unknown	[Similar to: Milbemycin A4 oxime; ΔMass: 18.1712 Da]	73.9	Full match	537.148	20.051
Unknown	[Similar to: 3-Methyl-5-(5,5,8a-trimethyl-2-methylene-7-oxodecahydro-1-naphthalenyl)pentyl acetate; ΔMass: -17.9354 Da]	78.7	Full match	366.202	30.283
Unknown	[Similar to: Obacunone; ΔMass: 0.0902 Da]	68.4	Full match	454.109	1.581
Unknown	[Similar to: Limonin; ΔMass: 60.0962 Da]	69.8	Full match	410.098	26.415
Unknown	[Similar to: (1α,2α,3β,5ξ,9ξ,18ξ)-1,2,3,19-Tetrahydroxyurs-12-en-28-oic acid; ΔMass: 64.1291 Da]	69.8	Full match	440.216	18.807
Unknown	[Similar to: Dodecyl gallate; ΔMass: 81.0854 Da]	80.5	Full match	257.124	1.656
Unknown	[Similar to: Enniatin B1; ΔMass: 299.2460 Da]	70.2	Full match	354.179	18.092
Unknown	[Similar to: L-Phenylalanine; ΔMass: -99.0684 Da]	72.4	Full match	264.147	12.925
Unknown	[Similar to: (+/-)12-HpETE; ΔMass: 0.0655 Da]	62.3	Full match	336.165	1.689
Unknown	[Similar to: 3,5-Dihydroxy-4-[3-(4-hydroxyphenyl)propanoyl]phenyl 6-O-(3,4,5-trihydroxybenzoyl)-β-D-glucopyranoside; ΔMass: 296.0919 Da]	64.9	Full match	292.056	1.594
Unknown	[Similar to: JWH 019 N-(6-hydroxyhexyl) metabolite; ΔMass: -18.0528 Da]	88.7	Full match	389.241	22.327

Unknown	[Similar to: Hesperetin; ΔMass: -212.0687 Da]	62.3	Full match	514.148	30.75
Unknown	[Similar to: N,N'-Bis(2,2,6,6-tetramethyl-4-piperidyl)-N,N'-diformylhexamethylenediamine; ΔMass: 121.1983 Da]	61.6	Full match	329.195	11.861
Unknown	[Similar to: Limonin; ΔMass: -25.9772 Da]	65.5	Full match	496.171	28.662
Unknown	[Similar to: (1S,4aR,5S)-5-[(3E)-5-Methoxy-3-methyl-5-oxo-3-penten-1-yl]-1,4a-dimethyl-6-methylenedecahydro-1-naphthaleneCarbohydrateoxylic acid; ΔMass: 42.0469 Da]	87	Full match	306.183	37.9
Unknown	[Similar to: 15-Deoxy-Δ12,14-prostaglandin J2-2-glycerol ester; ΔMass: -17.9338 Da]	81.3	Full match	408.174	2.923
Unknown	[Similar to: Palmitoyl sphingomyelin; ΔMass: -68.9752 Da]	60.1	Full match	771.543	53.834
Unknown	[Similar to: Thromboxane B2-biotin; ΔMass: 116.1809 Da]	68.4	Full match	564.237	53.715
Unknown	[Similar to: Dihomo-γ-linolenic acid methyl ester; ΔMass: -17.8417 Da]	70.4	Full match	338.113	36.01
Unknown	[Similar to: 2-Hydroxy-4,4,6,6-tetramethyl-2-(2-methyl-1-propen-1-yl)-3,5-dioxocyclohexyl 6-O-(3,4,5-trihydroxybenzoyl)-β-D-glucopyranoside; ΔMass: 236.1534 Da]	81.6	Full match	332.062	1.282
Unknown	[Similar to: Diphenyleneiodonium; ΔMass: -48.1885 Da]	73	Full match	326.148	19.585
Unknown	[Similar to: 3-O-β-D-Glucopyranosylandrographolide; ΔMass: -81.9862 Da]	84.2	Full match	594.248	50.157
Unknown	[Similar to: AL 8810; ΔMass: 38.0344 Da]	67.7	Full match	364.186	32.104
Unknown	[Similar to: Nervonic acid; ΔMass: 0.1689 Da]	75.2	Full match	366.181	36.311
Unknown	[Similar to: Cinchonine; ΔMass: -0.0463 Da]	89.8	Full match	294.22	45.4
Unknown	[Similar to: Tetrahydrocortisone; ΔMass: 0.0597 Da]	62.2	Full match	364.165	37.357
Unknown	[Similar to: Diphenyleneiodonium; ΔMass: -34.1729 Da]	70.7	Full match	312.132	15.013
Unknown	[Similar to: Diphenyleneiodonium; ΔMass: -82.1729 Da]	60.2	Full match	360.132	18.953
Unknown	[Similar to: Sucrose acetate isobutyrate; ΔMass: 534.2564 Da]	70.5	Full match	312.132	18.215
Unknown	[Similar to: Prostaglandin E2-1-glyceryl ester; ΔMass: 0.0615 Da]	78.6	Full match	426.2	14.471
Unknown	[Similar to: 2-{3-[(4E)-6-Acetoxy-2-hydroxy-6-methyl-3-oxo-4-hepten-2-yl]-6-(Carbohydrateoxymethyl)-2-hydroxy-3a,5a,9b-trimethyl-5-oxo-2,3,3a,4,5,5a,6,9,9a,9b-decahydro-1H-cyclopenta[a]naphthalen-7-yl}-2-methylpropanoic acid; ΔMass: 0.0559 Da]	78.3	Full match	590.253	51.839
Unknown	[Similar to: AL 8810; ΔMass: -19.9695 Da]	80	Full match	422.19	4.093
Unknown	[Similar to: 2-{4-[3-(5-Fluoro-1H-indol-3-yl)propyl]piperazino}-N-phenylacetamide fumarate; ΔMass: -114.0230 Da]	60.8	Full match	508.24	1.665
Unknown	[Similar to: Linoleic acid-biotin; ΔMass: -27.8978 Da]	77.1	Full match	548.242	51.513
Unknown	[Similar to: Metolachlor; ΔMass: -99.9739 Da]	93.9	Full match	383.108	11.71
Unknown	[Similar to: Propachlor OXA; ΔMass: -71.0735 Da]	62.1	Full match	278.163	18.937
Unknown	[Similar to: AL 8810; ΔMass: 38.0344 Da]	67.7	Full match	364.186	32.909
Unknown	[Similar to: {3-[(E)-2-(1,3-Benzodioxol-5-yl)vinyl]-2-oxiranyl}(1-piperidinyl)methanone; ΔMass: -67.9898 Da]	85	Full match	369.121	15.388
Unknown	[Similar to: 9S,13R-12-Oxophytodienoic acid; ΔMass: -18.0106 Da]	83.6	Full match	310.214	40.551
Unknown	[Similar to: Methyl (2E)-3-[5-acetoxy-9-(3-furyl)-4-hydroxy-3,3,5a,9a-tetramethyl-7-oxododecahydro[2]benzofuro[5,4-f]oxireno[d]isochromen-11b(1H)-yl]acrylate; ΔMass: 150.2570 Da]	65	Full match	393.974	1.098
Unknown	[Similar to: (2E)-2-({[(3aR,4R,6E,9S,10Z,11aS)-9-Acetoxy-6,10-dimethyl-3-methylene-2-oxo-2,3,3a,4,5,8,9,11a-octahydrocyclodeca[b]furan-4-yl]oxy}Carbohydrateonyl)-2-buten-1-yl (2E)-2-(hydroxymethyl)-2-butenoate; ΔMass: 175.0045 Da]	76.3	Full match	327.216	16.354
Unknown	[Similar to: Sorbitan monolaurate; ΔMass: -17.9491 Da]	83.4	Full match	364.185	4.045
Unknown	[Similar to: 4-[(1S,3aR,4S,6aR)-4-(3,4-Dimethoxyphenyl)tetrahydro-1H,3H-furo[3,4-c]furan-1-yl]-2-methoxyphenol; ΔMass:18.0622 Da]	65	Full match	354.095	14.728
Unknown	[Similar to: Diosgenin; ΔMass: -107.0366 Da]	71.2	Full match	521.35	47.845
Unknown	[Similar to: Tomatidine; ΔMass: -41.0479 Da]	74.3	Full match	456.393	46.371
Unknown	[Similar to: Sakuranin; ΔMass: -231.9815 Da]	90.7	Full match	680.119	10.353
Unknown	[Similar to: (1S,2S,8ξ,9β,16α,17ξ)-1-(β-D-Glucopyranosyloxy)-2,16,20-trihydroxy-9,10,14-trimethyl-11,22-dioxo-4,9-cyclo-9,10-secocholest-5-en-25-yl acetate; ΔMass: -123.8174 Da]	83.6	Full match	848.221	2.437
Unknown	[Similar to: 3-(5-Hydroxy-2,2,7,8-tetramethyl-6-oxo-7,8-dihydro-2H,6H-pyrano[3,2-g]chromen-10-yl)hexanoic acid; ΔMass: -137.9205 Da]	74.4	Full match	526.109	25.595
Unknown	[Similar to: Octadecanamine; ΔMass: -256.2040 Da]	66.5	Full match	525.512	51.918
Unknown	[Similar to: Schaftoside; ΔMass: -117.0151 Da]	72.8	Full match	681.163	30.535
Unknown	[Similar to: 12-Benzyl-3-isobutyl-6,9-diisopropyl-5,8-dimethyldodecahydro-1H,3H-pyrrolo[1,2-q][1,4,7,10,13,17]oxapentaazacyclononadecine-1,4,7,10,13,17(14H)-hexone; ΔMass: 96.9656 Da]	64.2	Full match	558.429	50.603
Unknown	[Similar to: Haplamine; ΔMass: -235.1608 Da]	66.4	Full match	492.266	1.673
Unknown	[Similar to: 2-Amino-1,3,4-octadecanetriol; ΔMass: -550.3630 Da]	67.7	Full match	867.656	43.13
Unknown	[Similar to: (2S,2'S,3R,3'R)-7'-(β-D-Glucopyranosyloxy)-5,5'-dihydroxy-2,2'-bis(4-hydroxyphenyl)-4,4'-dioxo-3,3',4,4'-tetrahydro-2H,2'H-3,3'-bichromen-7-yl β-D-glucopyranoside; ΔMass: 64.0805 Da]	93	Full match	802.146	1.938
Unknown	[Similar to: 9(Z),11(E),13(E)-Octadecatrienoic Acid methyl ester; ΔMass: -23.9612 Da]	98.6	Full match	316.201	44.17
Unknown	[Similar to: C-8 Ceramide-1-phosphate; ΔMass: -40.0034 Da]	70.3	Full match	545.357	31.595
Unknown	[Similar to: C-8 Ceramide-1-phosphate; ΔMass: -40.0033 Da]	68.2	Full match	545.357	32.396
Unknown	[Similar to: (4R,9β,23E)-2-(β-D-Glucopyranosyloxy)-16,20-dihydroxy-9,10,14-trimethyl-1,11,22-trioxo-4,9-cyclo-9,10-secocholesta-2,5,23-trien-25-yl acetate; ΔMass: -240.2105 Da]	89.5	Full match	958.567	55.899
Unknown	[Similar to: (2S,2'S,3R,3'R)-7'-(β-D-Glucopyranosyloxy)-5,5'-dihydroxy-2,2'-bis(4-hydroxyphenyl)-4,4'-dioxo-3,3',4,4'-tetrahydro-2H,2'H-3,3'-bichromen-7-yl β-D-glucopyranoside; ΔMass: -196.0001 Da]	66.9	Full match	1062.23	20.052
Unknown	[Similar to: Diosgenin; ΔMass: -107.1159 Da]	71.2	Full match	521.429	47.888
Unknown	[Similar to: Natamycin; ΔMass: 34.0268 Da]	78.5	Full match	631.278	33.671
Unknown	[Similar to: Octadecyl 3-(3,5-di-tert-butyl-4-hydroxyphenyl)propionate; ΔMass: -34.9506 Da]	81.7	Full match	565.421	25.774
Unknown	[Similar to: Chromomycin A3; ΔMass: 154.2255 Da]	87.9	Full match	1028.28	2.542
Unknown	[Similar to: C-8 Ceramide-1-phosphate; ΔMass: -40.0033 Da]	69.9	Full match	545.357	31.986
Unknown	[Similar to: Azithromycin; ΔMass: -85.0537 Da]	66.4	Full match	833.562	48.213
Unknown	[Similar to: Psychosine; ΔMass: 0.0727 Da]	84.7	Full match	461.263	23.777
Unknown	[Similar to: Tacrolimus; ΔMass: 170.1078 Da]	63.5	Full match	633.374	30.971
Unknown	[Similar to: Azithromycin; ΔMass: 140.1029 Da]	65.4	Full match	608.406	44.038
Unknown	[Similar to: Arctiin; ΔMass: 4.3033 Da]	89.4	Full match	529.907	1.066

*known fungal metabolite

Table S4 Secondary metabolites identified by LC FTICR-MS/MS (negative ion mode) and validated by predicted composition and/or ChemSpider, where unknown compounds were generated a similarity compound based on mzCloud simulation.

Class	Name	Formula	nzCloud Search	mzCloud Sim.	Predicted Compositions	ChemSpider	m/z	Molecular Weight	RT [min]
DiCarbohydrateoxylate	4-tert-Butyl 3-ethyl thiomorpholine-3,4-diCarbohydrateoxylate	C12 H21 N O4 S			Full match	Full match	274.11191	275.11918	34.495
Ester	Metilox	C18 H28 O3			Full match	Full match	291.19662	292.2039	44.07
Acid	ricinelaidic acid	C18 H34 O3			Full match	Full match	297.24358	298.25085	46.716
Other	14,14-Bis(ethylsulfanyl)dispiro[5.1.5.1]tetradecan-7-one	C18 H30 O S2			Full match	Full match	325.16573	326.17301	36.983
Alcohol	1,3-Bis{[3-(aminomethyl)benzyl]amino}-2-propanol	C19 H28 N4 O		65.6	Full match	Full match	327.21777	328.22505	37.274
fatty acid-taurine conjugate	N-tetradecanoyltaurine	C16 H33 N O4 S			Full match	Full match	334.20583	335.21311	43.76
Glycerol	Glycerol trivalerate	C18 H32 O6			Full match	Full match	343.21266	344.21994	30.183
Acid	DL-alpha-Hydroxybehenic acid	C22 H44 O3			Full match	Full match	355.32184	356.32912	51.636
quinoline	etisulergine	C19 H28 N4 O2 S			Full match	Full match	375.18484	376.19212	39.201
antibacterial	Actinonin	C19 H35 N3 O5			Full match	Full match	384.2505	385.25778	17.76
Phenol	2-{Bis[(4-phenyl-1,3-thiazol-2-yl)amino]methyl}phenol	C25 H20 N4 O S2			Full match	Full match	455.09871	456.10599	18.01
nucleoside	5'-O-{Hydroxy[(2-propylpentanoyl)oxy]phosphoryl}adenosine	C18 H28 N5 O8 P			Full match	Full match	472.15886	473.16614	12.282
Acid	Didecyl sebacate	C30 H58 O4				Full match	481.42643	482.43371	59.779
Fatty acid, derivative of cytarabine	Elacytarabine	C27 H45 N3 O6		97.9		Full match	506.3256	507.33288	51.127
Craboxylate	2-(6-Amino-1,3-dimethyl-2,4-dioxo-1,2,3,4-tetrahydro-5-pyrimidinyl)-2-oxoethyl 4-(2-thienylmethylene)-1,2,3,4-tetrahydro-9-acridineCarbohydrateoxylate	C27 H24 N4 O5 S			Full match	Full match	515.14112	516.1484	14.348
Flavone	5,7-Dihydroxy-2-nonacosyl-4H-chromen-4-one	C38 H64 O4			Full match	Full match	583.47366	584.48096	50.611
Pyridazine	2-[(4-Butyl-1-piperazinyl)methyl]-6-phenyl-3(2H)-pyridazinone	C19 H26 N4 O			Full match	Full match	325.20212	326.2094	35.655
Alcohol	1-{2-[2-(2-Phenoxypropoxy)propoxy]propoxy}-1-propanol	C18 H30 O5			Full match	Not the top hit	325.20212	326.20955	36.252
Acid	Diethyl diallylmalonate	C13 H20 O4		97.8	Full match	Full match	239.12894	240.13621	37.355
diCarbohydrateoxylic anhydride	(2-Dodecen-1-yl)succinic anhydride	C16 H26 O3		77.6	Full match	Full match	265.18096	266.18824	41.255
Acid	(E)-parinaric acid	C18 H28 O2		93.3	Full match	Full match	275.2017	276.20898	48.378
Alcohol	OCTOXYNOL-2	C18 H30 O3		72.4	Full match	Full match	293.21226	294.21954	44.064
Acid	4-Decylbenzenesulfonic acid	C16 H26 O3 S		75	Full match	Full match	297.15306	298.16033	46.214
Amine	5-Heptylbenzo[f]quinazoline-1,3-diamine	C19 H24 N4		70.7	Full match	Not the top hit	307.19155	308.19897	38.122
Amine	N-[1-(4-Benzyl-1-piperazinyl)-2-propanyl]-2-pyridinamine	C19 H26 N4		77.6	Full match	Full match	309.20717	310.21445	41.261
Acid	Undecylbenzenesulfonic acid	C17 H28 O3 S		80.2	Full match	Full match	311.16869	312.17597	47.826
serotonin 5-HT3 receptor	Granisetron	C18 H24 N4 O		70.6	Full match	Full match	311.18646	312.1938	38.866
Amine	1-Cyclohexyl-5-(1-piperidinylmethyl)-1H-benzimidazol-2-amine	C19 H28 N4		62.3	Full match	Not the top hit	311.22283	312.2301	39.523
Alcohol	1-{2-[2-(2-Phenoxypropoxy)propoxy]propoxy}-1-propanol	C18 H30 O5		94.6	Full match	Full match	325.20212	326.2094	37.402
Diol	(1S,2S)-1,2-Di[(2R)-1,4-dioxaspiro[4.5]dec-2-yl]-1,2-ethanediol	C18 H30 O6		73.3	Full match	Full match	341.19704	342.20432	32.898
cannabinoid	ADB-PINACA	C19 H28 N4 O2		72.9	Full match	Full match	343.21266	344.21994	32.003
dialkyl sulfosuccinates, are anionic surfactants	DIHEXYL SULFOSUCCINATE	C16 H30 O7 S		82	Full match	Full match	365.16404	366.17131	40.723
cannabinoid	ADB-CHMINACA	C21 H30 N4 O2		63.7	Full match	Full match	369.22839	370.23567	36.524
Amide	N-[3-(N'-CarbohydrateamimidoylCarbohydrateamimidamido)propyl]-N',N''-diphenylimidodiCarbohydrateonimidic diamide	C19 H26 N10		83.7	Full match	Full match	393.22845	394.23572	34.509
Amide	N-{2-[(CyclohexylCarbohydrateamoyl)(nitroso)amino]ethyl}-5-(2,4-dioxo-3,4-dihydro-1(2H)-pyrimidinyl)-3,4-dihydroxytetrahydro-2-furanCarbohydrateoxamide	C18 H26 N6 O8		85.3	Full match	Full match	453.17416	454.18143	11.063
Diol	Lexacalcitol	C29 H48 O4		84.2	Full match	Full match	459.34822	460.3555	51.664
Alcohol/phenol	4-tert-Octylphenol monoethoxylate	C16 H26 O2		93.2	Full match	Full match	499.3795	250.19336	47.23
Sugar	(1R,3S)-3-Glycoloyl-3,5,12-trihydroxy-10-methoxy-6,11-dioxo-1,2,3,4,6,11-hexahydro-1-tetracenyl beta-D-ribofuranoside	C26 H26 O13		88.7	Full match	Full match	545.13061	546.13788	23.064
Alcohol	OCTOXYNOL-2	C18 H30 O3		75.8	Full match	Full match	587.43214	294.21963	44.273
chlorophyll breakdown	pheophorbide b	C35 H34 N4 O6		83.4	Full match	Full match	605.24134	606.24862	50.123
Amine	N-(4-Isopropylphenyl)-5-[(4-methyl-1-piperazinyl)sulfonyl]-2-pyridinamine	C19 H26 N4 O2 S		62.7	Full match	Full match	373.16916	374.17643	37.773
Unknown	[Similar to: 16-Hydroxyhexadecanoic acid; ΔMass: 28.1040 Da]		Predicted	91.9	Full match		243.12384	244.13111	27.9
Unknown	[Similar to: Curcumin; ΔMass: 106.0782 Da]		Predicted	98.3	Full match		261.04053	262.0478	29.158
Unknown	[Similar to: 4-[(β-D-Glucopyranosyloxy)methyl]-6,7-dihydroxy-7-(hydroxymethyl)-1,4a,5,6,7,7a-hexahydrocyclopenta[c]pyran-1-yl 3-methylbutanoate; ΔMass: 238.1416 Da]		Predicted	84.1	Full match		239.05616	240.06344	16.282
Unknown	[Similar to: 11-Deoxy prostaglandin F2β; ΔMass: 70.1662 Da]		Predicted	60.9	Full match		267.07219	268.07947	1.259
Unknown	[Similar to: β-D-Fructose 6-phosphate ; ΔMass: -18.0106 Da]		Predicted	87.5	Full match		277.03304	278.04032	1.192
Unknown	[Similar to: 11(Z),14(Z),17(Z)-Eicosatrienoic acid; ΔMass: 0.0727 Da]		Predicted	68.4	Full match		305.17591	306.18319	37.938
Unknown	[Similar to: N-Feruloyloctopamine; ΔMass: 17.0417 Da]		Predicted	77	Full match		311.07731	312.08458	10.653

Unknown	[Similar to: Geniposide; ΔMass: 70.0749 Da]	Predicted	85.7	Full match	317.05481	318.06209	1.808
Unknown	[Similar to: Geniposide; ΔMass: 70.0749 Da]	Predicted	85.6	Full match	317.05481	318.06209	1.174
Unknown	[Similar to: 7-(Hydroxymethyl)-2,5a,11,11,13a-pentamethyl-3a,5a,6,8,12,13a,13b,13c-octahydrofuro[4,3,2-ij]pyrano[4',3':4,5]cyclohepta[1,2-f]isochromene-1,5,9,13(2H,11H)-tetrone; ΔMass: 117.9868 Da]	Predicted	76	Full match	325.18439	326.19166	49.549
Unknown	[Similar to: Docosahexaenoyl glycine; ΔMass: 57.0730 Da]	Predicted	78.8	Full match	327.18138	328.18865	34.324
Unknown	[Similar to: Bumetrizole; ΔMass: -14.9602 Da]	Predicted	95.5	Full match	329.06674	330.07402	33.271
Unknown	[Similar to: Gibberellin A4; ΔMass: 0.1091 Da]	Predicted	78	Full match	331.04602	332.05329	32.014
Unknown	[Similar to: (+/-)12-HpETE; ΔMass: -9.8389 Da]	Predicted	85.3	Full match	345.06172	346.069	34.944
Unknown	[Similar to: 2-[(2S,4aR,8aS)-2-Hydroxy-4a-methyl-8-methylenedecahydro-2-naphthalenyl]acrylic acid; ΔMass: -101.9802 Da]	Predicted	69	Full match	351.12975	352.13714	17.199
Unknown	[Similar to: Arachidic acid; ΔMass: -49.9042 Da]	Predicted	72.3	Full match	361.19978	362.20706	44.268
Unknown	[Similar to: 16-(Hexopyranosyloxy)-7-hydroxy-8,9-epoxypimaran-18-oic acid; ΔMass: 144.0421 Da]	Predicted	61.4	Full match	369.22839	370.23567	35.925
Unknown	[Similar to: Prostaglandin F1α; ΔMass: -17.9202 Da]	Predicted	64.5	Full match	373.16916	374.17643	37.384
Unknown	[Similar to: Hydroxykynurenine; ΔMass: -159.0281 Da]	Predicted	67	Full match	382.10057	383.10785	11.783
Unknown	[Similar to: (2E)-3-(4-Acetoxy-2,3-dihydroxy-2,5,5,8a-tetramethyldecahydro-1-naphthalenyl)acrylic acid; ΔMass: -32.0264 Da]	Predicted	71.1	Full match	385.22333	386.23061	37.328
Unknown	[Similar to: Calcitriol; ΔMass: 18.0620 Da]	Predicted	68.3	Full match	397.25974	398.26701	43.563
Unknown	[Similar to: Linoelaidic acid; ΔMass: -128.9699 Da]	Predicted	61.9	Full match	408.2029	409.21017	30.461
Unknown	[Similar to: Calcitriol; ΔMass: -7.9536 Da]	Predicted	70.3	Full match	423.27538	424.28266	49.381
Unknown	[Similar to: Anandamide (AEA); ΔMass: -77.9228 Da]	Predicted	66.9	Full match	424.19795	425.20523	21.707
Unknown	[Similar to: ARN1203; ΔMass: -59.9719 Da]	Predicted	69.6	Full match	438.25326	439.26054	32.35
Unknown	[Similar to: 3-Hydroxyurs-12-en-23-oic acid; ΔMass: 17.0539 Da]	Predicted	91	Full match	438.29918	439.30645	50.059
Unknown	[Similar to: 11-Deoxy prostaglandin F2β; ΔMass: -101.8476 Da]	Predicted	61	Full match	439.08602	440.09329	1.692
Unknown	[Similar to: 4a,7b-Dihydroxy-3-(hydroxymethyl)-9a-(isobutyryloxy)-1,1,6,8-tetramethyl-5-oxo-1a,1b,4,4a,5,7a,7b,8,9,9a-decahydro-1H-cyclopropa[3,4]benzo[1,2-E]azulen-9-yl (2E)-2-methyl-2-butenoate; ΔMass: 74.1821 Da]	Predicted	69.4	Full match	441.08296	442.09023	24.132
Unknown	[Similar to: Corchorifatty acid F; ΔMass: -116.0112 Da]	Predicted	79.3	Full match	443.22885	444.23613	36.013
Unknown	[Similar to: Corchorifatty acid F; ΔMass: -116.0112 Da]	Predicted	81.1	Full match	443.22885	444.23613	37.345
Unknown	[Similar to: Xanthohumol; ΔMass: -97.9254 Da]	Predicted	87.8	Full match	451.06488	452.07215	14.783
Unknown	[Similar to: Ascorbyl palmitate; ΔMass: -39.0239 Da]	Predicted	96.5	Full match	452.2784	453.28568	48.818
Unknown	[Similar to: Cucurbitacin I 2-O-β-D-glucopyranoside; ΔMass: 220.0873 Da]	Predicted	62.9	Full match	455.25131	456.25858	17.873
Unknown	[Similar to: 4H-1-Benzopyran-4-one, 6-β-D-glucopyranosyl-2,3-dihydro-5,7-dihydroxy-2-(4-hydroxyphenyl)-, (S)-; ΔMass: -23.9850 Da]	Predicted	67.7	Full match	457.09899	458.10627	15.781
Unknown	[Similar to: Afzelin; ΔMass: -29.9745 Da]	Predicted	78.7	Full match	461.07283	462.0801	23.955
Unknown	[Similar to: Lamalbid; ΔMass: -43.9454 Da]	Predicted	64.7	Full match	465.08053	466.0878	18.141
Unknown	[Similar to: Prostaglandin A1 ethyl ester; ΔMass: -110.9191 Da]	Predicted	89.6	Full match	474.17317	475.18045	13.649
Unknown	[Similar to: Methyl 2-{[2-O-(6-deoxy-α-L-mannopyranosyl)-β-D-glucopyranosyl]oxy}benzoate; ΔMass: -15.9385 Da]	Predicted	72.9	Full match	475.08849	476.09658	26.535
Unknown	[Similar to: Oleic acid; ΔMass: -197.0455 Da]	Predicted	96.6	Full match	478.29414	479.30142	49.378
Unknown	[Similar to: Docosahexaenoic acid; ΔMass: -153.0769 Da]	Predicted	96.6	Full match	480.30982	481.31709	50.836
Unknown	[Similar to: Oleic acid-biotin; ΔMass: 29.0432 Da]	Predicted	93.4	Full match	492.30984	493.31712	50.312
Unknown	[Similar to: 2-({4-[4-(2,4-dimethoxy-6-methylbenzoyloxy)-2-methyl-6-{[(2S,3R,4S,5S,6R)-3,4,5-trihydroxy-6-(hydroxymethyl)oxan-2-yl]oxy}benzoyloxy]-2-hydroxy-6-methylphenyl}formamido)propanoic acid; ΔMass: 235.9081 Da]	Predicted	66	Full match	492.31154	493.31882	45.138
Unknown	[Similar to: 2-(hydroxymethyl)-6-[(E)-4-(1,2,4-trihydroxy-2,6,6-trimethylcyclohexyl)but-3-en-2-yl]oxyoxane-3,4,5-triol; ΔMass: -89.8805 Da]	Predicted	70.3	Full match	495.09349	496.10077	12.706
Unknown	[Similar to: AUDA; ΔMass: -103.9384 Da]	Predicted	62.5	Full match	495.23506	496.24234	32.593
Unknown	[Similar to: Abietic acid; ΔMass: -198.1470 Da]	Predicted	70	Full match	499.36427	500.37155	51.65
Unknown	[Similar to: Arachidonic acid; ΔMass: -197.0456 Da]	Predicted	86.5	Full match	500.27851	501.28579	48.017
Unknown	[Similar to: 4H-1-Benzopyran-4-one, 6-β-D-glucopyranosyl-2,3-dihydro-5,7-dihydroxy-2-(4-hydroxyphenyl)-, (S)-; ΔMass: -70.0059 Da]	Predicted	92.6	Full match	503.11988	504.12725	25.647
Unknown	[Similar to: 1-O-(1,3,23,24,25-Pentahydroxy-28-oxo-9,19-cyclolanostan-28-yl)hexopyranose; ΔMass: 180.0631 Da]	Predicted	70.5	Full match	503.33813	504.3454	37.92
Unknown	[Similar to: Lariciresinol 4-O-glucoside; ΔMass: 16.1038 Da]	Predicted	89.9	Full match	505.09904	506.10631	26.531
Unknown	[Similar to: Quercetin-3β-D-glucoside; ΔMass: -42.0108 Da]	Predicted	61	Full match	505.09904	506.10631	25.376
Unknown	[Similar to: 4-Allyl-2-(β-D-glucopyranosyloxy)phenyl β-D-glucopyranoside; ΔMass: -43.9692 Da]	Predicted	66.2	Full match	517.13564	518.14292	22.468
Unknown	[Similar to: 4H-1-Benzopyran-4-one, 6-β-D-glucopyranosyl-2,3-dihydro-5,7-dihydroxy-2-(4-hydroxyphenyl)-, (S)-; ΔMass: -85.9894 Da]	Predicted	97	Full match	519.10344	520.11071	20.109
Unknown	[Similar to: 4H-1-Benzopyran-4-one, 6-β-D-glucopyranosyl-2,3-dihydro-5,7-dihydroxy-2-(4-hydroxyphenyl)-, (S)-; ΔMass: -85.9951 Da]	Predicted	97	Full match	519.10914	520.11642	20.107
Unknown	[Similar to: (+/-)12(13)-DiHOME; ΔMass: -205.8764 Da]	Predicted	96.6	Full match	519.11485	520.12212	19.986
Unknown	[Similar to: 4H-1-Benzopyran-4-one, 6-β-D-glucopyranosyl-2,3-dihydro-5,7-dihydroxy-2-(4-hydroxyphenyl)-, (S)-; ΔMass: -86.0008 Da]	Predicted	81.7	Full match	519.11485	520.12212	18.816
Unknown	[Similar to: Disperse red 1; ΔMass: -205.9843 Da]	Predicted	61.8	Full match	519.11491	520.12218	21.743

Unknown	[Similar to: 2-({4-[4-(2,4-dimethoxy-6-methylbenzoyloxy)-2-methyl-6-{[(2S,3R,4S,5S,6R)-3,4,5-trihydroxy-6-(hydroxymethyl)oxan-2-yl]oxy}benzoyloxy]-2-hydroxy-6-methylphenyl}formamido)propanoic acid; ΔMass: 207.8768 Da]	Predicted	72.7	Full match		520.34284	521.35011	47.913
Unknown	[Similar to: (9Z,12Z)-6,8-Dihydroxy-9,12-octadecadienoic acid; ΔMass: -210.0297 Da]	Predicted	66.3	Full match		521.25253	522.25981	40.866
Unknown	[Similar to: Taurolithocholic acid 3-sulfate; ΔMass: 33.0007 Da]	Predicted	60.9	Full match		529.25072	530.258	22.176
Unknown	[Similar to: cis-7-Hexadecenoic acid; ΔMass: -276.2068 Da]	Predicted	69.4	Full match		529.42408	530.43135	49.964
Unknown	[Similar to: Ascorbyl palmitate; ΔMass: -120.2026 Da]	Predicted	97.5	Full match		533.45712	534.4644	51.503
Unknown	[Similar to: (+/-)12(13)-DiHOME; ΔMass: -221.8714 Da]	Predicted	69.9	Full match		535.10984	536.11711	18.529
Unknown	[Similar to: 4-[(2S,3S,4S)-4-[4-(β-D-Glucopyranosyloxy)-3-methoxybenzyl]-3-(hydroxymethyl)tetrahydro-2-furanyl]-2-methoxyphenyl β-D-glucopyranoside; ΔMass: 148.1458 Da]	Predicted	75.4	Full match		535.10984	536.11711	19.386
Unknown	[Similar to: 3-[2-(1,3-Benzodioxol-5-yl)-7-methoxy-1-benzofuran-5-yl]-3-hydroxypropyl hexopyranoside;ΔMass: -35.9829 Da]	Predicted	94	Full match		540.3546	540.14607	1.786
Unknown	[Similar to: (2R,3E)-5-(3-Chloro-5-formyl-2,6-dihydroxy-4-methylphenyl)-3-methyl-1-[(1S,2R,6R)-1,2,6-trimethyl-3-oxocyclohexyl]-3-penten-2-yl acetate; ΔMass: -77.9078 Da]	Predicted	60	Full match		541.09709	542.10437	20.129
Unknown	[Similar to: (3β,5ξ,9ξ,18ξ)-28-Hydroxy-28-oxoolean-12-en-3-yl 6-deoxy-α-L-mannopyranosyl-(1->3)-[β-D-glucopyranosyl-(1->2)]-β-D-glucopyranosiduronic acid; ΔMass: 397.1256 Da]	Predicted	85.6	Full match		542.37025	543.37752	47.625
Unknown	[Similar to: (3β,6α,9ξ,12β,20Z)-3,12-Dihydroxydammara-20(22),24-dien-6-yl 2-O-(6-deoxy-α-L-mannopyranosyl)-β-D-glucopyranoside; ΔMass: 221.0934 Da]	Predicted	69.6	Full match		544.38603	545.3933	48.982
Unknown	[Similar to: 3'-Dephosphocoenzyme A; ΔMass: 141.0120 Da]	Predicted	79.6	Full match		545.12963	546.1369	15.276
Unknown	[Similar to: 3'-Dephosphocoenzyme A; ΔMass: 141.0110 Da]	Predicted	67	Full match		545.13061	546.13788	24.473
Unknown	[Similar to: Flavin adenine dinucleotide (FAD); ΔMass: 235.0617 Da]	Predicted	78.3	Full match		549.08819	550.09546	9.458
Unknown	[Similar to: ADB-PINACA; ΔMass: -205.9118 Da]	Predicted	75.4	Full match		549.1257	550.13298	19.854
Unknown	[Similar to: (9Z,12Z)-6,8-Dihydroxy-9,12-octadecadienoic acid; ΔMass: -238.2300 Da]	Predicted	78.4	Full match		549.4528	550.46008	56.115
Unknown	[Similar to: (4R,9β,23E)-2-(β-D-Glucopyranosyloxy)-16,20-dihydroxy-9,10,14-trimethyl-1,11,22-trioxo-4,9-cyclo-9,10-secocholesta-2,5,23-trien-25-yl acetate; ΔMass: 161.9949 Da]	Predicted	60.9	Full match		555.35423	556.36151	35.501
Unknown	[Similar to: 17-Octadecynoic acid; ΔMass: -276.2070 Da]	Predicted	63.2	Full match		555.43993	556.44721	50.737
Unknown	[Similar to: (1S)-1,5-Anhydro-2-O-(6-deoxy-α-L-mannopyranosyl)-1-[5,7-dihydroxy-2-(4-hydroxyphenyl)-4-oxo-4H-chromen-6-yl]-D-glucitol; ΔMass: 14.0152 Da]	Predicted	80.3	Full match		563.14112	564.1484	23.768
Unknown	[Similar to: (1S)-1,5-Anhydro-2-O-(6-deoxy-α-L-mannopyranosyl)-1-[5,7-dihydroxy-2-(4-hydroxyphenyl)-4-oxo-4H-chromen-6-yl]-D-glucitol; ΔMass: 14.0150 Da]	Predicted	80.3	Full match		563.14126	564.14853	23.896
Unknown	[Similar to: L-Glutathione oxidized; ΔMass: 45.0208 Da]	Predicted	75.5	Full match		566.12385	567.13113	9.52
Unknown	[Similar to: Phosphatidylinositol-1,2-dipalmitoyl; ΔMass: 238.2290 Da]	Predicted	87.1			571.28954	572.29682	48.997
Unknown	[Similar to: (9Z,12Z)-6,8-Dihydroxy-9,12-octadecadienoic acid; ΔMass: -260.2147 Da]	Predicted	75.5	Full match		571.43748	572.44476	53.649
Unknown	[Similar to: Arachidoyl ethanolamide; ΔMass: -219.1154 Da]	Predicted	63.7	Full match		573.45312	574.46039	54.843
Unknown	[Similar to: 10-Nitrolinoleate; ΔMass: -253.2063 Da]	Predicted	98	Full match		577.42428	578.43156	49.55
Unknown	[Similar to: (2R,3E)-5-(3-Chloro-5-formyl-2,6-dihydroxy-4-methylphenyl)-3-methyl-1-[(1S,2R,6R)-1,2,6-trimethyl-3-oxocyclohexyl]-3-penten-2-yl acetate; ΔMass: -115.0330 Da]	Predicted	69.7	Full match		578.22226	579.22954	12.045
Unknown	[Similar to: 9-Nitrooleate; ΔMass: -255.2221 Da]	Predicted	96.6	Full match		581.45576	582.46304	50.688
Unknown	[Similar to: Sorbitan monooleate; ΔMass: -158.1804 Da]	Predicted	98.1	Full match		585.4869	586.49418	51.852
Unknown	[Similar to: 4a,7b-Dihydroxy-3-(hydroxymethyl)-9a-(isobutyryloxy)-1,1,6,8-tetramethyl-5-oxo-1a,1b,4,4a,5,7a,7b,8,9,9a-decahydro-1H-cyclopropa[3,4]benzo[1,2-E]azulen-9-yl (2E)-2-methyl-2-butenoate; ΔMass: -75.9968 Da]	Predicted	70.8	Full match		591.26181	592.26909	52.316
Unknown	[Similar to: 16-Acetoxy-3-{[hexopyranosyl-(1->6)hexopyranosyl-(1->4)-2,6-dideoxy-3-O-methylhexopyranosyl]oxy}-14-hydroxycard-20(22)-enolide; ΔMass: 307.0572 Da]	Predicted	63.9	Full match		592.37097	593.37825	33.257
Unknown	[Similar to: 16-Acetoxy-3-{[hexopyranosyl-(1->6)hexopyranosyl-(1->4)-2,6-dideoxy-3-O-methylhexopyranosyl]oxy}-14-hydroxycard-20(22)-enolide; ΔMass: 307.0571 Da]	Predicted	63.1	Full match		592.37105	593.37833	33.699
Unknown	[Similar to: 7-(Hydroxymethyl)-2,5a,11,11,13a-pentamethyl-3a,5a,6,8,12,13a,13b,13c-octahydrofuro[4,3,2-ij]pyrano[4',3':4,5]cyclohepta[1,2-f]isochromene-1,5,9,13(2H,11H)-tetrone; ΔMass: -149.9808 Da]	Predicted	60.5	Full match		593.15193	594.1592	24.451
Unknown	[Similar to: 2-Methoxyestradiol; ΔMass: -293.9503 Da]	Predicted	75.1	Full match		595.13126	596.13854	23.426
Unknown	[Similar to: MEDICA 16; ΔMass: -256.1601 Da]	Predicted	91.4	Full match		597.42981	598.43708	50.688

Unknown	[Similar to: 1-O-[(2α,3β,5ξ,9ξ,18ξ,19α)-2,3,19,23-Tetrahydroxy-28-oxoolean-12-en-28-yl]-β-D-glucopyranose; ΔMass: 64.2609 Da]	Predicted	84.9	Full match	601.12972	602.137	1.818
Unknown	[Similar to: 1-O-[(2α,3β,5ξ,9ξ,18ξ,19α)-2,3,19,23-Tetrahydroxy-28-oxoolean-12-en-28-yl]-β-D-glucopyranose; ΔMass: 64.2513 Da]	Predicted	84.9	Full match	601.13932	602.1466	1.785
Unknown	[Similar to: Atorvastatin; ΔMass: -43.8961 Da]	Predicted	61.6	Full match	601.14187	602.14915	17.486
Unknown	[Similar to: 1-O-(1,3,23,24,25-Pentahydroxy-28-oxo-9,19-cyclolanostan-28-yl)hexopyranose; ΔMass: 82.1105 Da]	Predicted	78.3	Full match	601.29068	602.29795	38.404
Unknown	[Similar to: cis-5,8,11,14,17-Eicosapentaenoic acid; ΔMass: -300.2071 Da]	Predicted	71.7	Full match	601.42442	602.4317	49.474
Unknown	[Similar to: Sorbitan monooleate; ΔMass: -174.1545 Da]	Predicted	87	Full match	601.461	602.46828	51.85
Unknown	[Similar to: 5-[(1S,2R,4aR)-5-(Hydroxymethyl)-1,2,4a-trimethyl-1,2,3,4,4a,7,8,8a-octahydro-1-naphthalenyl]-3-methylpentanoic acid; ΔMass: -284.2120 Da]	Predicted	68.5	Full match	605.45556	606.46284	50.612
Unknown	[Similar to: 13,14-Dihydro-15-keto Prostaglandin A2 ; ΔMass: -276.2070 Da]	Predicted	64.8	Full match	609.41417	610.42145	44.25
Unknown	[Similar to: Urushenol; ΔMass: -296.1970 Da]	Predicted	63	Full match	613.44556	614.45283	45.559
Unknown	[Similar to: Oleyl anilide; ΔMass: -260.1844 Da]	Predicted	69.4	Full match	616.48031	617.48758	54.885
Unknown	[Similar to: cis-5,8,11,14,17-Eicosapentaenoic acid; ΔMass: -316.1812 Da]	Predicted	72.8	Full match	617.39851	618.40579	49.475
Unknown	[Similar to: (9Z,12Z)-6,8-Dihydroxy-9,12-octadecadienoic acid; ΔMass: 0.0000 Da]	Predicted	70.1	Full match	623.45353	312.2301	41.89
Unknown	[Similar to: (+/-)-Gingerol; ΔMass: -332.2123 Da]	Predicted	72.2	Full match	625.38817	626.39544	44.264
Unknown	[Similar to: 8(S)-Hydroxy-(5Z,9E,11Z,14Z)-eicosatetraenoic acid; ΔMass: -306.1965 Da]	Predicted	77.4	Full match	625.42441	626.43169	49.446
Unknown	[Similar to: Scutellarioside II; ΔMass: -120.0007 Da]	Predicted	63.4	Full match	627.15147	628.15874	34.36
Unknown	[Similar to: Anacardic acid; ΔMass: -282.1966 Da]	Predicted	91.6		629.45574	630.46302	50.539
Unknown	[Similar to: L-Glutathione oxidized; ΔMass: -19.1265 Da]	Predicted	61.8	Full match	630.27118	631.27846	33.648
Unknown	[Similar to:(2S,3Z,4S)-2-(β-D-Glucopyranosyloxy)-3-(2-{[(2E)-3-(4-hydroxyphenyl)-2-propenoyl]oxy}ethylidene)-4-(2-methoxy-2-oxoethyl)-3,4-dihydro-2H-pyran-5-Carbohydrateoxylic acid; ΔMass: -79.8674 Da]	Predicted	72.4	Full match	645.02372	646.031	19.658
Unknown	[Similar to: Arachidonic acid; ΔMass: -342.1966 Da]	Predicted	85.4	Full match	645.42958	646.43686	50.55
Unknown	[Similar to: R-Palmitoyl-(2-methyl) ethanolamide; ΔMass: -354.0808 Da]	Predicted	63.1	Full match	666.37157	667.37885	46.157
Unknown	[Similar to: Corchorifatty acid F; ΔMass: -350.2078 Da]	Predicted	67.1	Full match	669.42324	678.43278	35.665
Unknown	[Similar to: 7-(Hydroxymethyl)-2,5a,11,11,13a-pentamethyl-3a,5a,6,8,12,13a,13b,13c-octahydrofuro[4,3,2-ij]pyrano[4',3':4,5]cyclohepta[1,2-f]isochromene-1,5,9,13(2H,11H)-tetrone; ΔMass: 99.9583 Da]	Predicted	61.5	Full match	687.43339	344.22012	30.334
Unknown	[Similar to: 6-Deoxy-α-L-mannopyranosyl-(1->3)-[β-D-glucopyranosyl-(1->6)]-1-O-[3,27-dihydroxy-27,28-dioxolup-20(29)-en-28-yl]-β-D-glucopyranose; ΔMass: 290.2750 Da]	Predicted	83.8	Full match	701.19254	666.22311	1.987
Unknown	[Similar to: 16-(Hexopyranosyloxy)-7-hydroxy-8,9-epoxypimaran-18-oic acid; ΔMass: -191.8983 Da]	Predicted	79	Full match	705.16884	706.17611	19.377
Unknown	[Similar to: Xanthohumol; ΔMass: -354.0442 Da]	Predicted	68.3	Full match	707.18392	708.19089	14.787
Unknown	[Similar to: Scropheanoside I; ΔMass: -43.9184 Da]	Predicted	70.7	Full match	711.14272	712.15	18.634
Unknown	[Similar to: (4R,9β,23E)-2-(β-D-Glucopyranosyloxy)-16,20-dihydroxy-9,10,14-trimethyl-1,11,22-trioxo-4,9-cyclo-9,10-secocholesta-2,5,23-trien-25-yl acetate; ΔMass: -8.0304 Da]	Predicted	70	Full match	725.37956	726.38684	50.379
Unknown	[Similar to: 3-{[6-Deoxy-4-O-(β-D-glucopyranosyl)-α-L-mannopyranosyl]oxy}-14,16-dihydroxy-19-oxocard-20(22)-enolide; ΔMass: -23.8921 Da]	Predicted	66.5	Full match	735.21528	736.22277	27.604
Unknown	[Similar to: Crocin; ΔMass: 236.2184 Da]	Predicted	61.6	Full match	739.15306	740.16034	21.774
Unknown	[Similar to: 1-O-(1,3,23,24,25-Pentahydroxy-28-oxo-9,19-cyclolanostan-28-yl)hexopyranose; ΔMass: -79.7826 Da]	Predicted	93.9	Full match	763.18384	764.19111	1.977
Unknown	[Similar to: 1-O-(1,3,23,24,25-Pentahydroxy-28-oxo-9,19-cyclolanostan-28-yl)hexopyranose; ΔMass: -79.7916 Da]	Predicted	93.9	Full match	763.19275	764.20003	1.978
Unknown	[Similar to: 1-O-(1,3,23,24,25-Pentahydroxy-28-oxo-9,19-cyclolanostan-28-yl)hexopyranose; ΔMass: -884.0905 Da]	Predicted	71.4	Full match	783.2422	1568.49896	2.293
Unknown	[Similar to: (+/-)12(13)-DiHOME; ΔMass: -506.2899 Da]	Predicted	64.4	Full match	819.52832	820.5356	58.131
Unknown	[Similar to: (+/-)12(13)-DiHOME; ΔMass: -556.3776 Da]	Predicted	62.1	Full match	869.61605	870.62332	49.639
Unknown	[Similar to: 1-O-(1,3,23,24,25-Pentahydroxy-28-oxo-9,19-cyclolanostan-28-yl)hexopyranose; ΔMass: -143.8678 Da]	Predicted	75.7	Full match	873.27442	828.27624	2.292
Unknown	[Similar to: JWH 018 N-(5-hydroxypentyl) β-D-Glucuronide; ΔMass: -406.1897 Da]	Predicted	84.6	Full match	938.38741	939.39469	10.68
Unknown	[Similar to: (2S,2'S,3R,3'R)-7'-(β-D-Glucopyranosyloxy)-5,5'-dihydroxy-2,2'-bis(4-hydroxyphenyl)-4,4'-dioxo-3,3',4,4'-tetrahydro-2H,2'H-3,3'-bichromen-7-yl β-D-glucopyranoside; ΔMass: -196.0023 Da]	Predicted	82.8	Full match	1061.22192	1062.2292	20.111
Unknown	[Similar to: Chlorogenic acid; ΔMass: -162.0533 Da]	Predicted	82.9	Full match	515.14112	516.1484	12.28
Unknown	[Similar to: {(1R,2R)-2-[(2Z)-5-(Hexopyranosyloxy)-2-penten-1-yl]-3-oxocyclopentyl}acetic acid; ΔMass: 34.0782 Da]	Predicted	74.5	Full match	353.08786	354.09513	16.507
Unknown	[Similar to: [1-(3,4-dihydroxy-5-methoxyphenyl)-7-(3,4-dihydroxyphenyl)heptan-3-yl] acetate; ΔMass: -145.9495 Da]	Predicted	62.6	Full match	549.1257	550.13298	19.413
Unknown	[Similar to: 4H-1-Benzopyran-4-one, 6-β-D-glucopyranosyl-2,3-dihydro-5,7-dihydroxy-2-(4-hydroxyphenyl)-, (S)-; ΔMass: -86.0008 Da]	Predicted	97	Full match	519.11485	520.12212	20.108
Unknown	[Similar to: Corchorifatty acid F; ΔMass: -116.0112 Da]	Predicted	79.3	Full match	443.22885	444.23613	36.709

Unknown	[Similar to: 7(S),17(S)-Dihydroxy-8(E),10(Z),13(Z),15(E),19(Z)-docosapentaenoic acid; ΔMass: -3.9256 Da]	Predicted	82	Full match		365.16404	366.17131	40.938
Unknown	[Similar to: Calcitriol; ΔMass: 18.0620 Da]	Predicted	66	Full match		397.25974	398.26701	41.664
Unknown	[Similar to: (3β,5ξ,9ξ,18ξ)-28-Hydroxy-28-oxoolean-12-en-3-yl 6-deoxy-α-L-mannopyranosyl-(1->3)-[β-D-glucopyranosyl-(1->2)]-β-D-glucopyranosiduronic acid; ΔMass: 399.1413 Da]	Predicted	79.9	Full match		540.3546	541.36188	46.311
Unknown	[Similar to: Geniposide; ΔMass: -168.1551 Da]	Predicted	62.6	Full match		555.28482	556.2921	48.726
Unknown	[Similar to: Phosphatidylinositol-1,2-dipalmitoyl; ΔMass: 238.2290 Da]	Predicted	87.1	Full match		571.28954	572.29682	49.14
Unknown	[Similar to: 10-Nitrolinoleate; ΔMass: -269.1801 Da]	Predicted	91.6	Full match		593.39818	594.40545	49.549

Table S5 Lipid abundance by subclass in *Sphagnum fallax*.

Lipid	Replicate 1	Replicate 2	Replicate 3
Cer(d18:0/24:0)	551621.25	176054.5313	117811.7969
Cer(d18:1/24:0)	129682.5781	61852.17188	141658.8594
Cer(d18:0/14:0)	199475.6406	35165.92578	14977.09082
Cer(d18:0/15:0)	31480.85156	10464.02344	14666.90918
Cer(d18:0/16:0(2OH))	38634.36328	7726.437988	22469.17578
Cer(d18:0/16:0)	236248.25	57351.89844	65829.03906
Cer(d18:0/22:0)	192208.8125	77780.20313	73600.53125
Cer(d18:0/24:0)	413240.9063	127224.6406	173378.4688
Cer(d18:1/16:0(2OH))	21994.33984	6297.563965	15976.78613
Cer(d18:1/22:0(2OH))	801349.25	691499.875	958518
Cer(d18:1/22:0)	180106.5469	143066.2344	361140.875
Cer(d18:1/24:0(2OH))	2773811.25	2772395	2478581.5
Cer(d18:1/24:0)	81180.35156	67203.88281	195748.2188
Cer(d18:1/24:1(2OH))	490599.0938	380124.625	363215.625
Cer(d18:1/26:0(2OH))	737931.5	616676.125	491106.5625
Cer(d18:2/20:1)_A	277811.4688	256681.5	59686.45703
Cer(d18:2/20:1)_B	61701.57813	49071.4375	27233.10938
Cer(d19:1/17:1)	3689.587646	0	0
Cer(d20:1/16:0)	24125.00781	8833.336914	19677.64453
Cer(d20:1/17:0)	0	0	24318.30859
HexCer(d18:2/16:1)	12727.6416	9898.819336	9786.994141
HexCer(d18:2/20:1)	1269574.625	1159305.25	298324.2813
HexCer(d18:2/22:1)	109129.7031	59024.09766	159549.8906
PE_Cer(d34:1)	3578.196777	0	4307.551758
PE_Cer(d37:2)	4665.48584	7201.193848	4076.808838
PE_Cer(d38:2)	13485.9248	8962.349609	34633.17578
PA(16:0/18:1)	153271.8594	60186.79297	69406.92969
PA(16:0/18:2)	899106.9375	93601.07813	69067.875
PA(18:1/18:1)	218931.0938	166870.0625	278804.7813
PA(18:2/18:2)	487293.1563	45298.38281	32929.16797

PC(0:0/16:0)	79174.36719	94597.22656	92450.32031
PC(0:0/18:1)_A	60989.96484	47084.48438	103110.1953
PC(0:0/18:1)_B	175086	157349.3281	293992.5625
PC(16:0/18:1)	958793.3125	691093.8125	1733204.625
PC(16:0/18:2);PC(16:1/18:1)	760254.375	225697.2188	532901.625
PC(16:0/18:3)	102597.8984	20729.02734	46789.04688
PC(16:1/18:1)	1247147.875	405607.5	557069.5
PC(17:0/18:1)	106122.1875	59281.14063	253449.2188
PC(17:1/19:1)	645351.375	161024.9063	412633.6875
PC(18:1/18:1)	7492961.5	3548041.75	1.24E+07
PC(18:1/18:2)	549833.25	154005.9375	403780.0938
PC(18:1/19:1)	205794.1094	116597.3203	369571.2813
PC(18:2/18:2);PC(18:1/18:3)	286834.0313	55741.43359	198579.4688
PC(18:2/18:3)	48452.17578	7694.832031	45022.375
PE(15:0/15:0)	1152564	541980.0625	736320.75
PE(15:0/15:1)	191954.0938	58021.6875	111652.5078
PE(15:0/16:0)_A	195522.1406	44463.50391	33816.63672
PE(15:0/16:0)_B	128335.7578	63684.80078	159104.5313
PE(15:0/16:0)_C	357529.2813	182662.2344	367690.2188
PE(15:0/16:1)	694557.3125	463248.6563	1456643.5
PE(15:0/17:0)	485937.7188	169686.3125	582733
PE(15:0/17:1)_A	1113441	342576.6563	799126.125
PE(15:0/17:1)_B;PE(16:0/16:1)	424990	247771.3125	671996.4375
PE(15:0/18:1);PE(16:1/17:0);PE(16:0/17:1)_A	681621.9375	292439.125	884362
PE(16:0/17:1)_B	275433.8125	167288.8125	475759.0313
PE(16:0/18:1)	722540.5625	397754.8438	1069861.25
PE(16:0/18:2);PE(16:1/18:1)_B	1058336.125	199738.125	474414.75
PE(16:1/16:1)_A	255001.0781	171521.25	541963.8125
PE(16:1/16:1)_B	64376.54297	84595.21094	227813.5469
PE(16:1/18:1)_A	827744.8125	491601.3438	752219.6875
PE(17:0/17:0)	97602.65625	35148.74609	162644.7656
PE(17:1/17:1)	211851.1563	54315.85938	142262.0938
PE(18:1/18:1)	2188925.5	1917905.5	2182657.25

PE(18:1/18:2)	220071	51068.125	277174.0625
PE(18:1/19:1)	485867.125	187304.2031	309222.5938
PG(15:0/15:0)	214306.75	159534.5469	142874.6719
PG(15:0/16:0)	94125.77344	84276.20313	102879.2344
PG(16:0/16:1)	110121.5469	105538.1797	184770.0156
PG(16:0/18:1)	296796.5625	331004.8125	419390.5
PG(16:1/16:1)	32995.50781	30192.19336	72471
PG(16:1/18:1)	410991.25	194433.4688	495570.3125
PG(18:1/18:1)	1116097.125	1004116.188	2075775.375
PI(15:0/15:0)	480700.875	323361.3125	596154.3125
PI(16:0/18:2)	445517.0625	40812.01563	51941.71484
DG(18:0/0:0/18:0)	459858.0938	580958.6875	1064340.375
DG(18:1/0:0/18:1)	156323.8594	552198.1875	165284.4219
DG(20:0/0:0/20:0)	175129.8594	234890.4688	136396.9688
DGDG(16:1/18:1)_A	62260.30078	63052.16016	24887.37891
DGDG(16:1/18:1)_B;DGDG(16:0/18:2)	925102.1875	101395.4297	23368.58203
DGDG(19:1/19:1)	353478.75	98311.72656	227655.0625
SQDG(16:0/18:1)	77006.30469	511743.5938	63026.83594
DGTSA(16:0/16:0)	322148.6875	463897.7188	350988.375
DGTSA(16:0/16:1)	1905562	2124836.75	3125968.5
DGTSA(16:0/18:1)	2257745.25	4393849	839831.125
DGTSA(16:0/18:2)	643683.625	2129550	784985.8125
DGTSA(16:0/18:3)	82088.9375	291949.25	141327.2969
DGTSA(16:1/16:1)	208527.0313	333033.7188	1861389.625
DGTSA(16:1/18:1)	920083.75	944477.8125	1381702.375
DGTSA(17:0/17:0)	148680.5625	87825.48438	199475.4219
DGTSA(18:1/18:1)	4508082.5	3399777.75	1219671.125
DGTSA(18:1/18:2)	226123.2031	283795.5	125096.0547
TG(15:0/16:0/16:0)	86656.86719	101385.0234	88599.46875
TG(16:0/16:0/16:0)	283626.0938	346335.0625	211995.8906
TG(16:0/16:0/18:0)	118180.3203	144605.5469	160464.8594
TG(16:0/16:0/18:1)	85208.42969	128538.8359	131654.9844
TG(16:0/16:1/18:1)	107476.8047	109270.2109	97742.09375

TG(16:0/18:0/18:0)	136956.375	162519.8594	235497.7188
TG(16:0/18:1/18:1)	240592.9531	333269.4063	377987
TG(16:0/18:2/18:2)	194884.1719	111930.8125	140562.0469
TG(16:1/16:1/16:1)	18252.59766	18814.94531	637178
TG(16:1/18:1/18:1);TG(16:0/18:1/18:2)	150963.6719	191053.5469	224747.7969
TG(18:0/18:0/18:0)	78839.85156	115665.6328	210080.2813
TG(18:0/18:0/18:1)	18854.88867	23620.41016	32193.37305
TG(18:0/18:1/18:1)	50106.56641	69163.21875	86787.14844
TG(18:1/18:1/18:1)	336842.5313	398117.2188	546538.625
TG(18:1/18:1/18:2)	200659.6406	157072.6719	234035.3906
TG(18:2/18:2/18:2)	210745.8125	87811.92188	102788.1484
TG(54:5)	167253.4844	120994.4219	216986.8438
CL(18:1/18:1/18:1/18:1)	199009.5313	189486.2344	221818.2813
MIPC(t18:0/24:0(2OH))	213820.4531	311964.7813	232646.3906
CoQ9	18375.65234	0	0
Unknown_542.48_16.2_NL299,256,244_PI267	272688.3438	206178.0938	114733.2969
Unknown_554.52_19.2_NL299,256,244_PI267	124063.9063	38263.65625	38489.14844
Unknown_570.51_18.3_NL299,256,244,60_PI267	530704.125	158984.8906	181811.0469
Unknown_598.54_20.5_NL299,256,244,60_PI267	730441.25	282262.125	478578.5938
Unknown_610.58_22.6_NL299,256,244_PI267	431044.3125	320632.9063	324364.9063
Unknown_624.59_23.1_NL299,256,244_PI267	127073.6953	90041.875	69598.67188
Unknown_626.57_22.1_NL299,256,244_PI267	2249446	2100797.5	1062175.875
Unknown_638.61_23.6_NL299,256,244_PI267	4270233.5	3490966	4392270
Unknown_652.63_24.1_NL299,256,244_PI267	2481024.75	2909478.25	2029677.875
Unknown_654.6_23.2_NL299,256,244_PI267	1.76E+07	1.63E+07	1.57E+07
Unknown_654.6_23.6_NL299,256,244,60_PI267	246605.5469	218435.2344	131022.0234
Unknown_664.62_23.6_NL299,256,244_PI267	433404.0625	352983.7188	109199.0781
Unknown_666.64_24.5_NL299,256,244_PI267	1.12E+07	1.18E+07	1.01E+07
Unknown_668.62_23.7_NL299,256,244_PI267	1.98E+07	2.10E+07	1.56E+07
Unknown_680.62_23.2_NL299,256,244_PI267	1.85E+07	1.53E+07	8564022
Unknown_680.66_24.9_NL299,256,244_PI267	1915274	2097513	1459173.375
Unknown_682.63_24.2_NL299,256,244_PI267	7.12E+07	6.97E+07	4.40E+07
Unknown_682.64_245_NL299,256,244,60_PI267	994880.6875	692066.3125	467151.125

Unknown_694.6_17.2_NL299,256,244_PI267	1702818	1500387.125	471619.125
Unknown_694.64_23.7_NL299,256,244_PI267	1690376.25	1304834.875	693834
Unknown_694.67_25.3_NL299,256,244_PI267	4629823	5038023	4504919
Unknown_696.61_19.1_NL299,256,244_PI267	368649.2813	590767	103688.1094
Unknown_696.62_16.7_NL299,256,244_PI267	1495866.25	1404209.625	330359.4375
Unknown_696.62_17.9_NL299,256,244_PI267	124353.4141	144317.7969	74978.67969
Unknown_696.65_24.6_NL299,256,244_PI267	2.10E+07	2.46E+07	1.51E+07
Unknown_696.65_24.9_NL299,256,244,60_PI267	383381.375	304352.4688	126361.3672
Unknown_698.63_17.8_NL299,256,244_PI267	222825.6094	228064.0469	135548.8906
Unknown_708.65_24.1_NL299,256,244_PI267	725467.1875	517251.5	361226.3438
Unknown_708.69_25.6_NL299,256,244_PI267	220780.6719	208638.3438	147545.7813
Unknown_710.67_25.0_NL299,256,244_PI267	3.03E+07	3.04E+07	1.57E+07
Unknown_710.67_25.3_NL299,256,244,60_PI267	1282018	620999.625	292329.1875
Unknown_724.68_25.4_NL299,256,244_PI267; Unknown_724.68_25.3_NL299,256,244,60_PI267	2641066	4263953	1785345.375
Unknown_738.7_25.7_NL299,256,244_PI267; Unknown_738.7_25.7_NL299,256,244,60_PI267	742626	1033843	585952.8125
Unknown_748.72_25.9_NL299,256,244_PI267	130162.0781	58772.08594	91444.25781

Table S6 Fungal metabolites identified through positive mode LC-FTICR-MS/MS and confirmed using predicted compositions (number/intensity of isotopes and fragments) and ChemSpider (mass).

Formula	Molecular Weight	DeltaMass [ppm]	Rank	# Matched Iso.	# Missed Iso.	# Matched Frag.	SFit [%]	Pattern Cov. [%]	MS Cov. [%]	MSMS Cov. [%]	In ChemSpider
$C_{28}H_{42}O$	394.32357	0.31	1	3	0	6	78.23923465	99.07	95.8	0.98	TRUE
$C_{28}H_{44}O$	396.33922	0.18	1	3	0	11	86.86693875	99.05	100	46.99	TRUE
$C_{23}H_{32}N_4O$	380.25761	-3.34	1	2	0	14	96.9904231	95.7	100	9.11	TRUE

Sfit%: Spectral similarity score between theoretical and measured isotope pattern in %
Pattern Cov. [%]: matched intensity percentage of the theoretical pattern
MS Cov. [%]: matched intensity percentage of the relevant portion of the MS
MSMS Cov. [%]: matched intensity percentage of the MSMS scan

Table S7 List of masses and formulas of the seventeen (15 masses + 2 isomers) antimicrobial compounds (starred) and first neighbor compounds.

Exact Mass	Formula	Class
193.0717374	$C_6H_{12}O_4N_2$	Amino Sugars
355.1246479	$C_9H_{14}O_6$	Amino Sugars
357.1038584	$C_8H_{14}O_5N_2$	Amino Sugars
379.067037	$C_{14}H_{23}O_8N_3$	Amino Sugars
387.0721166	$C_{13}H_{21}O_9N_3$	Amino Sugars
318.998362	$C_{18}H_{35}O_{10}P_1$	Amino Sugars
192.059463	$C_7H_{12}O_5$	Carbohydrates
205.0717849	$C_6H_{10}O_6$	Carbohydrates
209.0666914	$C_6H_{12}O_6$	Carbohydrates
235.0459365*	$C_7H_{12}O_6$	Carbohydrates
239.0772359	$C_6H_{12}O_6$	Carbohydrates
247.0571834	$C_7H_{14}O_6$	Carbohydrates
313.1140401	$C_8H_{14}O_6$	Carbohydrates
323.0984523	$C_7H_{14}O_5N_2$	Carbohydrates
339.0933233	$C_7H_{12}O_7$	Carbohydrates
343.1246379	$C_7H_{14}O_7$	Carbohydrates
369.103871	$C_8H_{12}O_7$	Carbohydrates
383.1195138	$C_7H_{12}O_6N_2$	Carbohydrates
203.0349931	$C_9H_{14}O_7$	Carbohydrates
205.0142593	$C_8H_{14}O_6N_2$	Carbohydrates
219.0298863	$C_8H_{12}O_8$	Carbohydrates
245.0455524	$C_8H_{16}O_8$	Carbohydrates
271.0248137	$C_8H_{12}O_7N_2$	Carbohydrates
299.0561325	$C_9H_{14}O_7N_2$	Carbohydrates
369.0616315	$C_{10}H_{16}O_7N_2$	Carbohydrates
371.0408193	$C_9H_{16}O_8N_2$	Carbohydrates
183.0662848	$C_{11}H_{19}O_8N_1$	Carbohydrates
195.0662804	$C_{11}H_{18}O_9$	Carbohydrates

207.0298975	$C_{11}H_{22}O_9$	Carbohydrates
209.0455461	$C_{10}H_{20}O_{10}$	Carbohydrates
267.0874026	$C_{11}H_{22}O_{10}$	Carbohydrates
293.0666622	$C_{12}H_{20}O_{10}$	Carbohydrates
309.0980221	$C_{11}H_{20}O_{11}$	Carbohydrates
327.0721735	$C_{12}H_{20}O_{11}$	Carbohydrates
335.0773203	$C_{12}H_{22}O_{11}$	Carbohydrates
337.0564712	$C_{11}H_{22}O_{11}$	Carbohydrates
341.0878559	$C_{12}H_{24}O_{11}$	Carbohydrates
351.0721819	$C_{13}H_{22}O_{11}$	Carbohydrates
357.2070672	$C_{13}H_{24}O_{11}$	Carbohydrates
361.0564428	$C_{12}H_{22}O_{12}$	Carbohydrates
371.0983501	$C_{12}H_{24}O_{12}$	Carbohydrates
407.0983238	$C_{13}H_{22}O_{12}$	Carbohydrates
461.1089064	$C_{14}H_{24}O_{12}$	Carbohydrates
475.1244481	$C_{14}H_{21}O_{10}N_3$	Carbohydrates
503.0830073	$C_{14}H_{22}O_{13}$	Carbohydrates
827.2671467	$C_{15}H_{28}O_{15}S_1$	Carbohydrates
315.0933154	$C_{18}H_{32}O_{16}$	Carbohydrates
285.1707195	$C_{18}H_{32}O_{17}$	Carbohydrates
217.0717494*	$C_9H_6O_3$	Aromatics
293.0878748	$C_{11}H_8O_4$	Aromatics
297.1191396	$C_{10}H_6O_5$	Aromatics
359.1194858	$C_{11}H_8O_5$	Aromatics
519.15653	$C_{13}H_{10}O_4$	Aromatics
257.0455802	$C_{13}H_{10}O_5$	Aromatics
269.045561	$C_{12}H_8O_6$	Aromatics
297.0405054	$C_{14}H_{10}O_5$	Aromatics
327.0510648*	$C_{15}H_{10}O_5$	Aromatics
331.0459969	$C_{14}H_8O_6$	Aromatics
574.1070897	$C_{15}H_8O_6$	Aromatics

163.0400496	$C_{16}H_{12}O_5$	Aromatics
175.0400777*	$C_{15}H_{10}O_6$	Aromatics
179.0349601	$C_{14}H_{10}O_6$	Aromatics
199.0611913	$C_{16}H_{10}O_6$	Aromatics
207.0662803	$C_{16}H_{12}O_6$	Aromatics
209.0819518	$C_{15}H_{10}O_7$	Aromatics
247.0612297	$C_{17}H_{12}O_6$	Aromatics
251.0561448	$C_{16}H_{10}O_7$	Aromatics
269.0666579*	$C_{16}H_{12}O_7$	Aromatics
302.1478995	$C_{17}H_{12}O_7$	Aromatics
309.1092268	$C_{16}H_{10}O_8$	Aromatics
313.0718048	$C_{16}H_{12}O_8$	Aromatics
331.1915793	$C_{18}H_{14}O_7$	Aromatics
354.0911508	$C_{18}H_{12}O_8$	Aromatics
359.077319	$C_{18}H_{14}O_8$	Aromatics
401.0878887	$C_{19}H_{14}O_8$	Aromatics
409.077594	$C_{18}H_{12}O_9$	Aromatics
227.2016733	$C_{33}H_{10}O_1$	Aromatics
189.0404665	$C_{29}H_{22}O_{12}$	Aromatics
360.1412213*	$C_9H_8O_3$	Lignins
175.061179	$C_9H_{10}O_3$	Lignins
177.0404262	$C_8H_8O_4$	Lignins
191.0560821	$C_{10}H_8O_3$	Lignins
205.0829773	$C_{10}H_{10}O_3$	Lignins
207.0510129	$C_9H_8O_4$	Lignins
219.0510213*	$C_9H_{10}O_4$	Lignins
219.062255	$C_9H_{12}O_4$	Lignins
233.0779251	$C_{10}H_8O_4$	Lignins
261.0728216	$C_{10}H_{12}O_4$	Lignins
275.0884432	$C_9H_{10}O_5$	Lignins
279.0834093	$C_9H_{12}O_5$	Lignins

299.0983773	$C_{11}H_{10}O_4$	Lignins
327.0933048	$C_{10}H_8O_5$	Lignins
341.1088999	$C_{11}H_{12}O_4$	Lignins
342.1123127	$C_{10}H_{10}O_5$	Lignins
353.1089677	$C_{11}H_{14}O_4$	Lignins
390.1153927	$C_{11}H_{10}O_5$	Lignins
397.0988421	$C_{11}H_{12}O_5$	Lignins
479.1075776	$C_{10}H_{10}O_6$	Lignins
229.050651	$C_{11}H_{10}O_6$	Lignins
283.0248291	$C_{13}H_{12}O_5$	Lignins
283.0612712	$C_{12}H_{10}O_6$	Lignins
285.0404745	$C_{12}H_{12}O_6$	Lignins
301.03541	$C_{13}H_{12}O_6$	Lignins
311.0561328	$C_{13}H_{14}O_6$	Lignins
313.0353808	$C_{12}H_{12}O_7$	Lignins
315.0510932	$C_{13}H_{16}O_6$	Lignins
329.0303897	$C_{12}H_{14}O_7$	Lignins
341.0666935	$C_{15}H_{12}O_5$	Lignins
355.0459238	$C_{15}H_{14}O_5$	Lignins
357.0615844	$C_{14}H_{12}O_6$	Lignins
165.05568	$C_{13}H_{16}O_7$	Lignins
181.0506155	$C_{15}H_{12}O_6$	Lignins
191.0349845	$C_{14}H_{14}O_7$	Lignins
197.0455407	$C_{14}H_{16}O_7$	Lignins
205.0506365	$C_{14}H_{18}O_7$	Lignins
221.0455607	$C_{17}H_{22}O_4$	Lignins
223.0612106	$C_{15}H_{12}O_7$	Lignins
225.0405016	$C_{15}H_{18}O_7$	Lignins
237.0404961	$C_{14}H_{18}O_6N_2$	Lignins
249.0404684	$C_{14}H_{16}O_8$	Lignins
263.0561463	$C_{17}H_{14}O_6$	Lignins

267.051049	$C_{14}H_{18}O_8$	Lignins
271.0611897	$C_{17}H_{16}O_6$	Lignins
275.0561235	$C_{16}H_{14}O_7$	Lignins
287.0561049	$C_{15}H_{16}O_8$	Lignins
295.0823535	$C_{14}H_{14}O_9$	Lignins
297.098032	$C_{15}H_{18}O_8$	Lignins
303.0510091	$C_{14}H_{16}O_9$	Lignins
311.0772526	$C_{20}H_{26}O_4$	Lignins
313.0929523	$C_{20}H_{28}O_4$	Lignins
315.0874357	$C_{16}H_{14}O_8$	Lignins
317.0666768	$C_{16}H_{16}O_8$	Lignins
323.0772615	$C_{15}H_{14}O_9$	Lignins
325.0565305	$C_{16}H_{18}O_8$	Lignins
325.0928479	$C_{15}H_{16}O_9$	Lignins
333.0615915*	$C_{15}H_{18}O_9$	Lignins
337.0929568	$C_{18}H_{16}O_7$	Lignins
339.0722318	$C_{15}H_{20}O_9$	Lignins
343.0823144	$C_{17}H_{14}O_8$	Lignins
343.103509	$C_{16}H_{16}O_9$	Lignins
345.0616158*	$C_{16}H_{18}O_9$	Lignins
353.0878437	$C_{15}H_{18}O_9$	Lignins
355.1035213	$C_{16}H_{20}O_9$	Lignins
361.0928905*	$C_{22}H_{30}O_4$	Lignins
367.1035379*	$C_{18}H_{16}O_8$	Lignins
375.0722177	$C_{17}H_{16}O_8$	Lignins
381.0828243	$C_{17}H_{14}O_9$	Lignins
381.119072	$C_{18}H_{18}O_8$	Lignins
391.1034161	$C_{17}H_{16}O_9$	Lignins
397.0776512	$C_{17}H_{20}O_9$	Lignins
403.1035078	$C_{16}H_{18}O_{10}$	Lignins
417.0826889	$C_{16}H_{20}O_{10}$	Lignins

431.0982753	$C_{18}H_{16}O_9$	Lignins
447.0931667	$C_{17}H_{16}O_{10}$	Lignins
448.0965079	$C_{17}H_{18}O_{10}$	Lignins
461.0724226	$C_{18}H_{22}O_9$	Lignins
463.0882186	$C_{20}H_{18}O_8$	Lignins
464.0914359	$C_{19}H_{16}O_9$	Lignins
477.1038163	$C_{19}H_{20}O_9$	Lignins
479.0829786	$C_{17}H_{18}O_{11}$	Lignins
521.1299802	$C_{20}H_{18}O_9$	Lignins
535.1091738	$C_{20}H_{20}O_9$	Lignins
535.2547352	$C_{19}H_{20}O_{10}$	Lignins
609.1460064	$C_{18}H_{18}O_{11}$	Lignins
697.3076282	$C_{20}H_{18}O_{10}$	Lignins
421.0754282	$C_{21}H_{18}O_{10}$	Lignins
159.0106407	$C_{21}H_{20}O_{10}$	Lignins
230.9823526	$C_{20}H_{18}O_{11}$	Lignins
389.1313623	$C_{21}H_{18}O_{11}$	Lignins
389.1677707*	$C_{21}H_{20}O_{11}$	Lignins
427.1091875	$C_{20}H_{20}O_{11}$	Lignins
443.1404231	$C_{21}H_{18}O_{12}$	Lignins
449.1087029	$C_{22}H_{22}O_{11}$	Lignins
473.1087691*	$C_{21}H_{20}O_{12}$	Lignins
489.1036046	$C_{20}H_{20}O_{12}$	Lignins
491.1193323	$C_{21}H_{22}O_{12}$	Lignins
517.1408667	$C_{22}H_{20}O_{12}$	Lignins
549.1248049	$C_{23}H_{24}O_{11}$	Lignins
625.1407534	$C_{22}H_{22}O_{12}$	Lignins
633.1716422	$C_{21}H_{20}O_{13}$	Lignins
165.0404723	$C_{23}H_{20}O_{13}$	Lignins
361.2231692	$C_{24}H_{26}O_{13}$	Lignins
374.15687	$C_{28}H_{38}O_{10}$	Lignins

375.2025083	$C_{24}H_{24}O_{14}$	Lignins
447.1516988	$C_{28}H_{40}O_{10}$	Lignins
243.0622831	$C_{27}H_{30}O_{16}$	Lignins
376.0997395	$C_{34}H_{50}O_{15}$	Lignins
292.1037929*	$C_{12}H_{24}O_2$	Lipids
503.1616699	$C_{14}H_{28}O_2$	Lipids
286.0438533	$C_{16}H_{32}O_2$	Lipids
167.034971*	$C_{18}H_{36}O_2$	Lipids
255.2329955	$C_{16}H_{26}O_3N_2S_4$	Lipids
175.0724223		None
161.0244221		None
421.065647		None
283.0823445		None
363.0721933		None
465.103834		None
475.0880989		None
199.1703505		None
283.2643036		None
282.0844094		None
358.210548		None
435.093153		None
505.0985727		None
163.0248118		None
165.0404263		None
341.0391213		None
403.0646847		None
173.0455304		None
357.0827356		None
359.0984298		None
503.1405556		None
217.0829966	$C_5H_8O_6$	Other

362.1205088	$C_5H_{10}O_6$	Other
441.1894954	$C_5H_{10}O_6$	Other
265.071753	$C_{10}H_{18}O_{11}$	Other
273.0768461	$C_{10}H_{20}O_{11}$	Other
329.1758675	$C_9H_{15}O_{10}N_2P_1$	Other
533.2392795	$C_{12}H_{21}O_{13}P_1$	Other
177.0557188	$C_{15}H_{26}O_5$	Proteins
385.0929013	$C_{18}H_{34}O_7$	Proteins
429.0826718	$C_{15}H_{25}O_8N_3$	Proteins
433.0775173	$C_{18}H_{32}O_8$	Proteins
419.0750533	$C_{20}H_{32}O_7S_2$	Proteins
179.0560924	$C_7H_{10}O_5$	Tannins
233.0666842	$C_7H_{10}O_6$	Tannins
247.024836	$C_9H_{12}O_6N_2$	Tannins
360.0806097	$C_{15}H_{18}O_{10}$	Tannins
369.0827806	$C_{15}H_{20}O_{10}$	Tannins
445.0775595	$C_{13}H_{19}O_{10}N_3$	Tannins
313.0776887	$C_{21}H_{28}O_{14}$	Tannins
