# Supplementary material for: Untargeted metabolomic profiling of Sphagnum fallax reveals novel antimicrobial metabolites
Source: Plant Direct. 2019 Nov 12;3(11):e00179. doi: 10.1002/pld3.179 (PMC6848953; doi:10.1002/pld3.179)
Supplement: Supplementary file 2 [file PLD3-3-e00179-s002.docx]

Response to reviewer

ANSWER: We would like to thank reviewer # 2 for his fast turnaround of the paper. As suggested, we went ahead and responded to each point separately here as well as in the main manuscript draft. Thanks again for your time!

Reveiwer # 2

I appreciate the effort of the Authors to revise the manuscript, indeed most of the issues have been discussed/corrected. Below please find a few questions/comments still not addressed: - the method underlying the quantification of the metabolites should be explained, chemical shifts of signal used for respective elucidations should be provided in the supplemental data or Table 2 - this issue has not been addressed by the Authors in the revised manuscript.

ANSWER: We thank the reviewer again for taking time to provide their comments and suggestions. Please note that we already added the chemical shifts of signal used for respective elucidations in Table S1 in the first revised draft. However, we might have failed to include the table in our first R2R document. The following sentence is currently in the materials and methods section of the paper: “A list of the average chemical shifts per identified metabolite is included in Table S1”.

We also included it here below:

Table 1. Table S1. A list of the average chemical shifts per identified metabolite is listed in Table is included in Table S1. m- multiplet, s- singlet, d, doublet, t-triplet, q- quartet, dd- doublet of doublets, dt-doublet of triplets

Also we added few sentences that explains how quantification was done. These were included in the materials and methods section of the paper. They are also listed below. “The 1D 1H NMR spectra of all samples were processed, assigned and analyzed using Chenomx NMR Suite 8.3 that combines chemistry and advanced algorithms to identify and quantify metabolites in NMR spectra with an extensive library of spectral models of metabolites (https://www.chenomx.com). **In contrast to other analytical methods, only one internal standard is necessary for NMR analysis. Here, quantification was achieved through the use of an internal standard 4,4-dimethyl-4-silapentane-1-sulfonic acid (DSS) that was used as chemical shift references for the calibration of the NMR data (at δ 0.0 ppm) as well as an internal standard for quantitation where spectral intensities of metabolites were quantified relative to the DSS internal standard**. Candidate metabolites present in each of the complex mixtures were determined by matching the chemical shift, J-coupling and intensity information of experimental NMR signals screened against the NMR signals of standard metabolites in the Chenomx library (along with HMDB and some custom metabolite additions totaling ~ 1000 metabolites). A list of the average chemical shifts per identified metabolite is included in Table S1” - the 'methanol story' - I cannot agree with the Authors that methanol must have been totally removed from the samples, in my opinion a short statement explaining the possibility that traces of methanol might originate from the extraction procedure should be added in the Results

Answer: We thank the reviewer for his comment. We also agree with her/his statement. As such, we added a sentence to the materials and discussion section that explains the possibility that traces of methanol might originate from the extraction procedure. The following sentence reads as follows: “The sample was vacuum dried then reconstituted in deionized H2O. Even though vacuum drying would remove the majority of methanol in the sample, traces of methanol might remain in the sample after drying. Thus our reported methanol concentration might be an overestimation of the true concentration of methanol in the plant extract” - the Authors should explain in the manuscript the methodology 'how the extraction efficiency was estimated' or provide the appropriate reference

Answer: We thank the reviewer for his/her comment. Two references has been added on line 331. Title - please correct the plant Latin name - instead of Sphagnum Fallax it should read Sphagnum fallax

This has been done. Thank you!
